# Mapping of QTLs for Seed Phorbol Esters, a Toxic Chemical in *Jatropha curcas* (L.)

**DOI:** 10.3390/genes8080205

**Published:** 2017-08-18

**Authors:** Kitiya Amkul, Kularb Laosatit, Prakit Somta, Sangrea Shim, Suk-Ha Lee, Patcharin Tanya, Peerasak Srinives

**Affiliations:** 1 Department of Agronomy, Faculty of Agriculture at Kamphaeng Saen, Kasetsart University, Nakhon Pathom 73140, Thailand; heartless_gg@hotmail.com (K.A.); pigirosa@hotmail.com (K.L.); agrprt@ku.ac.th (P.T.); agrpss@ku.ac.th (P.S.); 2 Department of Plant Science and Research Institute of Agriculture and Life Sciences, College of Agriculture and Life Sciences, Seoul National University, Seoul 151-921, Korea; kev8305@gmail.com (S.S.); sukhalee@snu.ac.kr (S.-H.L.)

**Keywords:** phorbol esters, Jatropha, physic nut, quantitative trait loci, QTL, seed toxin

## Abstract

Jatropha (*Jatropha curcas* L.) is an oil-bearing plant that has potential to be cultivated as a biodiesel crop. The seed cake after oil extraction has 40–50% protein that can be used in animal feeds. A major limitation in utilizing the cake is the presence of phorbol esters (PE), a heat-tolerant toxic chemical. To identify the quantitative trait loci (QTLs) for PE, we constructed a genetic linkage map from an F_2_ population of 95 individuals from a cross “Chai Nat” × “M10” using 143 simple sequence repeat (SSR) markers. M10 is low in seed PE while Chai Nat is high. Seeds from each F_2_ individual were quantified for PE content by high performance liquid chromatography. A single marker analysis revealed five markers from linkage group 3 (LG3) and nine markers from LG8 associated with seed PE. Inclusive composite interval mapping identified two QTLs, each on LG3 (*qPE3.1*) and LG8 (*qPE8.1*) responsible for the PE. *qPE3.1* and *qPE8.1* accounted for 14.10%, and 15.49% of total variation in seed PE, respectively. Alelle(s) from M10 at *qPE3.1* increased seed PE, while at *qPE8.1* decreased seed PE. *qPE3.1* is a new loci for PE, while *qPE8.1* is the same locus with that reported recently for PE.

## 1. Introduction

Jatropha or physic nut (*Jatropha curcas* L.) (2n = 2x = 22) is an oil-bearing plant that has potential to be cultivated as a non-edible oil crop for producing biodiesel. Although Jatropha seed has 30–44% oil with high percentage of monounsaturated oleic and polyunsaturated linoleic acid [1,2] this crop has not been fully domesticated due to several limitations including low seed yield, non-synchronous maturity, and the presence of toxins in seeds. The presence of this chemical in seed cakes after oil extraction from the seeds prevent the use of the cake which contain high protein (40–50%) as raw material for animal feed industry [3]. The principal seed toxins in Jatropha are phorbol esters (PE) and curcin [4,5]. PE, a tumor inducing substance, is tetracyclic tiglian diterpenoids. PE is found in most Jatropha accessions (called toxic Jatropha). Six forms of PE have been isolated from Jatropha in which all of them are likely to be derived from the same phorbol backbone [4,5]. Despite the interest in PE, its biosynthesis pathway is still poorly understood. Nonetheless, geranylgeranyl diphosphate (GGPP) synthase, casbene synthase, terpene synthases, terpene hydroxylase and acyltransferase are believed to implicate in PE biosynthesis [6,7,8,9,10]. A single knockdown of genes for GGPP synthase, terpene synthase and casbene synthases by RNA interference (RNAi) technique reduced up to 80% of PE content in the leaves of Jatropha, while a double knockdown of two of these genes reduced PE content in the leaves to less than 15% of control toxic Jatropha [10]. However, some accessions from Mexico and Guatemala have no or low PE in seeds (non-toxic Jatropha) [11,12]. The low PE seeds are consumed as snack or sweets. The high and low PE seeds can be distinguished only by seed analysis with high performance liquid chromatography (HPLC).

Breeding for non-toxic cultivar(s) is a major objective in Jatropha breeding programs, although there are a few reports on genetics of seed PE in Jatropha [12,13]. Plant breeders are looking for an inexpensive and quick method to differentiate high and low PE seed Jatropha such as through molecular markers, provided that the location(s) of gene(s) controlling seed PE content is known. Recently, King [12] reported that seed PE in Jatropha is controlled by a single dominant gene without xenia effect. They also located a quantitative trait locus (QTL) controlling seed PE onto a genetic linkage map. Although the QTL is closely to several simple sequence repeat (SSR) markers, such the QTL should be validated before using for marker-assisted selection. Therefore, in this study, our objective was to we identify and validate QTLs for PE for Jatropha using different source of non-toxic cultivar.

## 2. Materials and Methods 

### 2.1. Plant Materials and DNA Extraction

An F_2_ population of 95 individuals was developed by self-pollinating an F_1_ hybrid derived from a cross between “Chai Nat” (hereafter called “CN”) and “M10” as male and female parents, respectively. Homozygosity in both CN and M10 is higher than 99.0%. M10 is a non-toxic cultivar originated from Mexico possessing low seed PE, while CN is a toxic cultivar of Thailand possessing high PE. Previously, the PE content of M10 and CN was reported to be 0.05 and 1.62 mg/g, respectively [13]. During the self-pollination, flowers of the F_1_ hybrid were covered with pollen-proof bags to prevent pollen contamination from other plants. The F_2_ population was grown in an experimental field of Kasetsart University, Kamphaeng Saen Campus, Thailand during March 2014 to June 2015. The mature seeds from each plant were individually harvested. Genomic DNA of each plant was extracted from young leaves following a cetyltrimethylammonium bromide (CTAB) method described by Tanya et al. [13]. 

### 2.2. Quantification of Phorbol Esters Content in Seeds

**Figure 1:**
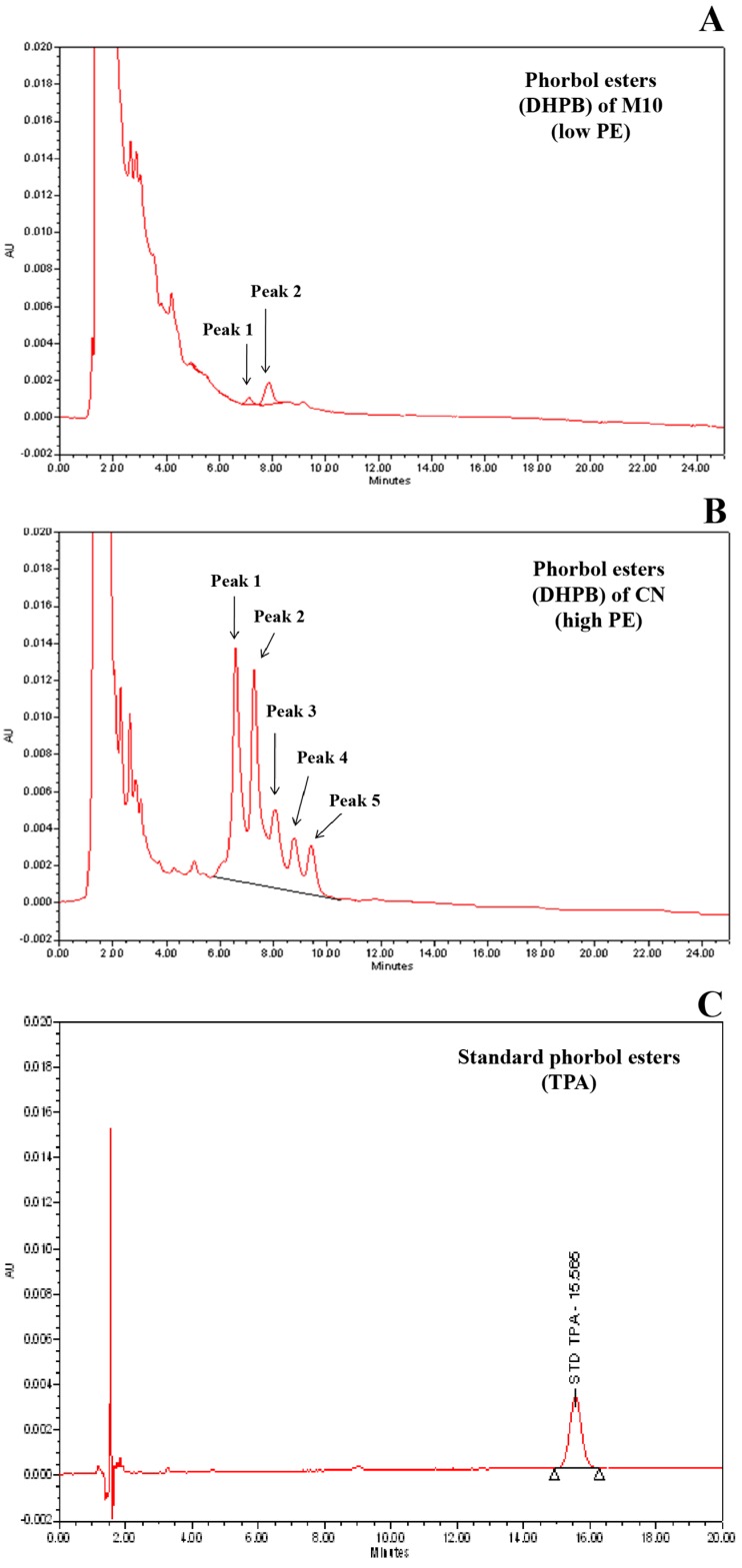
High Pressure Liquid Chromatography (HPLC) chromatogram of phorbol esters in seed of M10 (non-toxic Jatropha) (**A**) and Chai Nat (toxic Jatropha) (**B**) and of phorbol 12-myristate 13-acetate (TPA) (**C**). DHPB: 12-deoxy-16-hydroxyphorbol; PE: phorbol esters; CN: Chai Nat.

The mature seeds were used to analyze for PE content following the method described by Haas and Mittelbach [14] with minor modifications. Briefly, kernels were grinded into fine powder. A sample of four grams from each plant was extracted for PE by methanol (analytical grade) for five hours in a soxhlet apparatus (Buchi Universal Extraction System B-811, Buchi, Flawil, Switzerland). The methanol was then removed from the solvent using a rotary evaporator (Buchi Rotavapor R-205, Buchi, Flawil, Switzerland) to obtain dried extract. The dried extract was dissolved in 25 mL methanol (HPLC grade). The PE content was determined using an HPLC (Waters 600 HPLC system, Waters, Milford, MA, USA) equipped with a reverse phase C-18 column and photodiode array detector. The column temperature was maintained at 25 °C and the flow rate was kept at 1 mL min^−1^. The mobile phase was acetonitrile and water in 80/20 ratio. The PE peaks appeared between 6 and 10 min were detected and integrated at 280 nm. The phorbol 12-myristate 13-acetate (TPA) (Sigma Aldrich, St. Louis, MO, USA) was used as an external standard, which appeared between 20 and 22 min (Figure 1C). The areas under the PE peaks were summed and converted to standard PE equivalent by taking its peak area and concentration. The quantification was conducted twice for each plant. The average value of seed PE content of each plant was used for QTL analysis.

### 2.3. SSR Marker Analysis

One thousand and eighty SSR markers from the reports of King et al. [12]; Laosatit et al. [8,14]; Tanya et al. [15] and Wang et al. [16] were used to screen for polymorphism between M10 and CN. Polymerase chain reaction (PCR), electrophoresis, and DNA bands visualization were carried out following Laosatit et al. [14]. Polymorphic SSR markers were used to analyze DNA of the F_2_ plants. 

### 2.4. Linkage Map Construction and QTL Analysis

For each marker in the F_2_ population, plants showing homozygous DNA bands of CN and M10 parents were scored as “2” and “0”, respectively, while plants showing heterozygous DNA bands were scored as “1”. All the markers were checked for segregation distortion using chi-squared test. A genetic linkage map was constructed using software QTL IciMapping 4.0 (Chinese Academy of Agricultural Sciences, Beijing, China) [17]. The markers were grouped using a minimum log of the odds (LOD) of 4. The markers were put in order by “RECORD” and “SARF” functions. Map distance was calculated based on Kosambi’s mapping function. Linkage group was named following the linkage map reported by King et al. [12] using common marker(s).

SSR markers associated with seed PE content were determined by likelihood ratio test (LRT) method implemented in the software QTL IciMapping 4.0 using significant LOD threshold of 2.5. Locations of the QTLs controlling seed PE were determined by inclusive composite interval mapping (ICIM) [18] implemented in the QTL IciMapping 4.0. Significant LOD score for QTLs were obtained from 3000 permutation test at *p* = 0.05.

### 2.5. Identification of Physical Locations of Markers Flanking QTLs and Genes Associated with QTL

Primer sequences of the markers locating around QTL regions detected for PE were subjected to BLASTN analysis (NCBI—National Center for Biotechnology Information, Bethesda, MD, USA) against the *Jatropha curcas* Database (JCDB) (http://jcdb.xtbg.ac.cn) to identify the physical location and explore potential candidate genes that may implicate in PE biosynthesis.

## 3. Results

### 3.1. Variation of Seed PE in Parents and F_2_ Population

**Figure 2:**
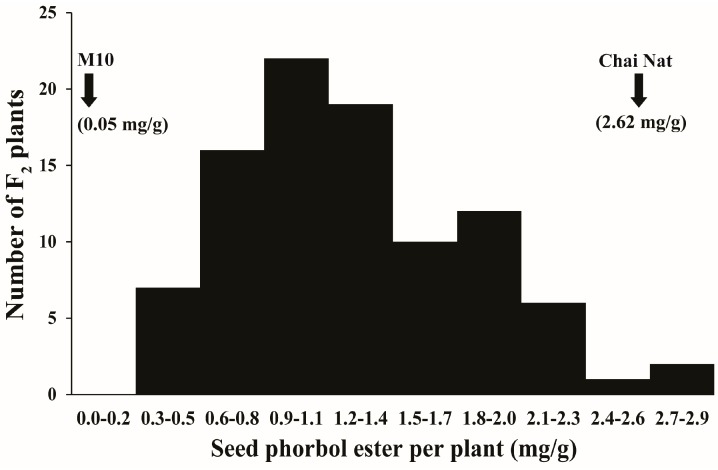
Frequency distribution of phorbol esters content in seeds (mg per gram) of the 95 F_2_ plants derived from the cross Chai Nat × M10. PE was determined by HPLC method.

CN and M10 possessed a large difference in seed PE, with 2.62 and 0.05 mg/g, respectively, which is about 52-fold difference in seed PE content. In addition, M10 and CN parents exhibited different types/forms of PE. CN showed five forms (Figure 1A) while M10 showed two forms which were also present in CN (Figure 1B). Seed PE of the F_2_ plants distributed continuously skewing toward M10 ranging from 0.30 to 2.89 mg/g with a mean of 1.33 mg/g (Figure 2). This suggests that the seed PE content in Jatropha is a quantitative trait.

### 3.2. SSR Polymorphism and Linkage Map

Out of 1080 SSR markers screened in CN and M10, only 742 markers were able to be amplified from DNA of the CN and/or M10 (Table S1). All except a few of the amplifiable markers showed single DNA band in each parent. Out of 742 amplifiable markers, 143 markers (19.27%; 100 genic and 43 genomic SSRs) showed polymorphism. This indicated a low genetic difference between CN and M10.

**Figure 3:**
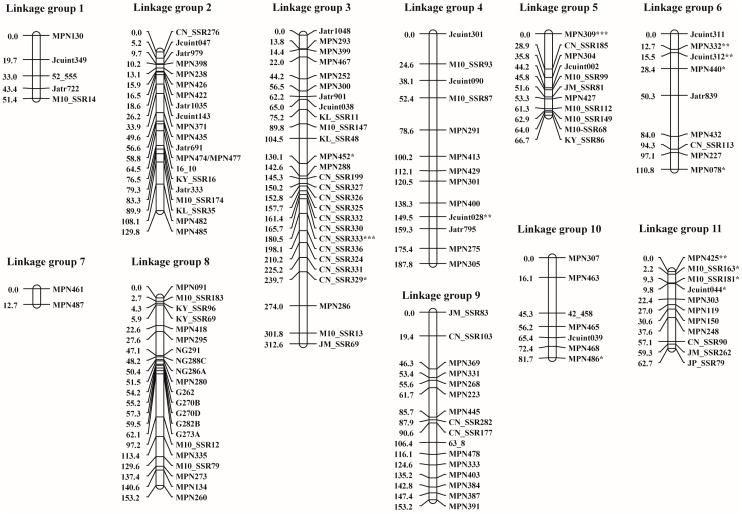
The genetic linkage map developed from 95 F_2_ plants derived from the cross Chai Nat × M10 using 143 simple sequence repeat (SSR) markers. Genetic distance was shown in centimorgan unit. Linkage groups were named as per King et al. [6]. Markers showing significant deviation from the expected segregation ratio at 0.05, 0.01, and 0.001 probability levels are marked with *, **, and ***, respectively.

All the 143 polymorphic markers were used to analyze the F_2_ population. Among these markers, 14 of them showed segregation distortion. The linkage map of the F_2_ population developed from the 143 polymorphic SSR markers comprised 11 linkage groups (LG) spanning a total length of 1322.6 cM (Figure 3). The number of markers per LG ranged from 2 (LG7) to 27 (LG2 and LG3) with an average of 14.3. LG7 and LG3 were the shortest and the longest linkages with the length of 12.7 and 312.6 cM, respectively. The average distance between the adjacent markers was 9.3 cM. The distorted markers were distributed onto 5 LGs (Figure 3). Clusters of 4 and 3 distorted markers were found on LG11 and LG6, respectively. 

### 3.3. QTL Controlling Seed PE in Jatropha

**Figure 4:**
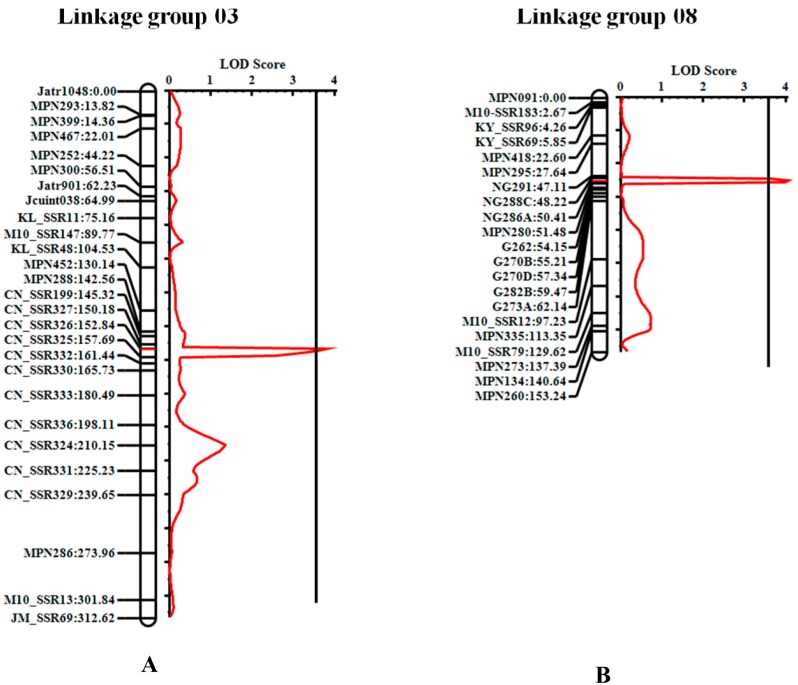
Log of the odds (LOD) graph of the quantitative trait loci (QTL) *qPE3.1* (**A**) and *qPE8.1* (**B**) controlling seed phorbol esters content detected in the F_2_ population of Chai Nat × M10 by inclusive composite interval mapping.

QTL mapping using LRT method revealed that five markers on LG3 and nine on LG8 associated with PE in the seeds. These markers accounted for 11.74% (CN_SSR199 on LG3) to 16.99% (NG286A on LG8) of the variation of seed PE in the F_2_ population (Table 1). QTL mapping using inclusive composite interval mapping (ICIM) method detected two QTLs, one each on LG3 and LG8, and they were named as *qPE3.1* and *qPE8.1*, respectively (Table 2 and Figure 4). *qPE3.1* and *qPE8.1* accounted for 14.10% and 15.49% of the total PE variation in the F_2_ population, respectively. *qPE3.1* showed additive effect of −0.09 mg/g and dominant effect of −0.41 mg/g. This QTL showed overdominance effect (d/a = 4.56) (Table 2)*.* Interestingly, allele from M10 at this QTL increased PE content. *qPE8.1* showed additive effect of 0.31 mg/g and dominant effect of −0.02 mg/g. Allele from M10 at this QTL decreased the PE content. Altogether, *qPE3.1* and *qPE8.1* accounted 29.59% of the total PE variation in the population.

### 3.4. Physical Location of the PE QTLs and Annotated Genes in the QTL Regions

**Figure 5:**
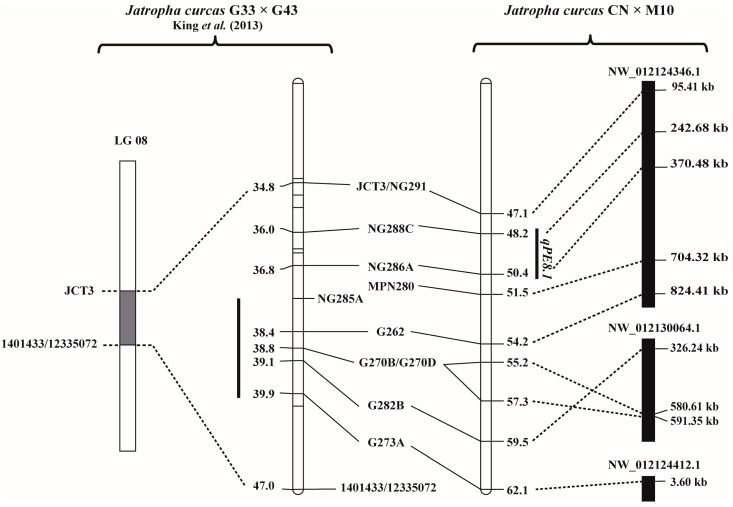
Comparative map illustrating the position of *qPE8.1* for seed phorbol esters content in the F_2_ population of Chai Nat × M10 identified in this study (right) and the location of locus controlling seed phorbol esters in the F_2_ population of G33 × G43 reported by King et al. [12] (left). Lines connect common markers between the two maps. Small solid bars along the maps indicate confidence intervals of the locus detected for seed phorbol esters. The QTL for PE reported by King was initially mapped between markers JCT3 and 1401433/12335072 by considering the PE as a quantitative trait. The PE locus was then fine mapped between markers NG285A and G273A by considering PE as a qualitative trait. Three solid bars along the map of Chai Nat × M10 represent Jatropha reference sequence from *Jatropha curcas* Database (JCDB).

BLASTN analysis against the JCDB revealed that the markers CN_SSR326 and CN_SSR325 that flank the PE QTL *qPE3.1* on LG3 were at the positions 1069652 and 1074572 of the sequence NW012124225.1, respectively. There was only one annotated gene in this region, LOC105634082. LOC105634082 encodes an uncharacterized protein. The markers NG288C and G286A that flank PE QTL *qPE8.1* on LG8 were at the positions 242687 and 370487 of the sequence NW012130064.1, respectively (Figure 5). There were 16 annotated genes between these two markers (Table 3).

## 4. Discussion

King et al. [12] reported that the presence of PE is a qualitative trait controlled by a single dominant gene. They identified a single major QTL controlling the trait. On the contrary, we found in this study that the presence of PE is a quantitative trait (Figure 1). Recently, Ng [10] reported that down regulate single gene expression of GGPP synthase, terpene synthase and casbene synthases reduced up to 80% of PE content in the leaves of Jatropha, while a double down regulate of two genes reduced PE content in the leaves to 85%. This suggested that PE contents in Jatropha is a quantitative trait. Thus, our results agreed with that report of Ng [10]. The contrasting findings between our study and that of King et al. [12] may stem from the fact that the jatropha germplasm used by King et al. [12] was different from that used in this study. The non-toxic Jatropha (G43) used by them contained no PE, while the one (M10) used in this study contained very low PE. G43 was from Guatemala, while M10 was from Mexico. In addition, the extraction methods used to determine PE in the two studies were different. It is also worth noting that non-toxic (M10) and the toxic (CN) Jatropha parents used in this study exhibited different types/forms of PE (Figure 1A,B). Regardless the above argument on germplasm and quantifying methods, the location of *qPE8.1* controlling seed PE detected in our study was similar to the QTL conferring seed PE reported by King et al. [12]. *qPE8.1* was located between markers NG288C and NG286A which is within the genomic region reported by them (Figure 5). This suggested that the QTLs controlling seed PE for Jatropha identified in these two studies are the same locus. Thus, this QTL was validated and can be used for marker-assisted selection (MAS).

*qPE3.1* is a new QTL controlling seed PE content in Jatropha. In contrast to *qPE8.1*, the allele contributed by CN at *qPE3.1* reduced PE content (Table 2). In addition, *qPE3.1* showed strong dominant effect or overdominance effect (Table 2). This suggests that this QTL is possibly a cluster of linked loci for PE that resulting in pseudo-overdominance effect. Nonetheless, *qPE3.1* in combination with *qPE8.1* will be useful for MAS to develop new Jatropha line(s) possessing low seed PE and for map-based cloning of the gene(s) controlling PE.

In this study, two QTLs were detected explaining a total of 29.59% of PE variation in the F_2_ Jatropha population. The low total variance explained by the QTLs suggested that the PE is highly affected by environments. However, since the F_2_ population comprised only 95 plants, it is possible that QTLs with small effects controlling PE (if any exist) were not detected. In addition, effect of QTL detected under small population size is likely to be overestimated, while its position cannot be located accurately [19]. Therefore, number and effects of the QTLs for PE found in this study must be considered carefully before using in MAS and map-based cloning.

Based on BLASTN analysis against the JCDB, there were 17 annotated genes between the flanking markers of *qPE3.1* and *qPE.81* (Table 3). However, none of these genes encodes enzyme that possibly involves in PE biosynthesis such as GGPP synthase, casbene synthase, terpene synthases, terpene hydroxylase and acyltransferase [6,7,8,9,10]. Nevertheless, the marker flanking *qPE3.1* and *qPE.81* will be useful for MAS and these genome regions can be used as target for fine mapping to identify gene controlling PE content in Jatropha.

Tanya et al. [15] studied diversity of jatropha using genic SSR markers and reported that markers MPN006, MPN007, MPN008, MPN016 and MPN046 were able to classify toxic and non-toxic Jatropha accessions. Nonetheless, in this study, none of these markers showed polymorphism between toxic and non-toxic Jatropha parents used in this study. This result indicated that these markers cannot be used for classifying high and low toxic Jatropha germplasm, at least between CN and M10.

## Figures and Tables

**Table 1:** Simple sequence repeat (SSR) markers associated with seed phorbol esters content in F_2_ population of Chai Nat × M10 detected by likelihood ratio test method. Log of the odds (LOD) value of 2.5 was used as a significant threshold.

LG *^a^*	Marker Name	*R*^2^ (%) *^b^*	Position	LOD Score
3	CN_SSR199	11.74	145.32	2.58
3	CN_SSR327	12.25	150.18	2.70
3	CN_SSR326	15.88	152.84	3.57
3	CN_SSR330	11.67	165.73	2.56
3	CN_SSR336	12.88	198.11	2.84
8	NG291	14.20	47.11	3.16
8	NG288C	13.06	48.22	2.89
8	NG286A	16.99	50.41	3.84
8	MPN280	16.40	51.48	3.70
8	G262	13.55	54.15	3.00
8	G270B	15.69	55.21	3.52
8	G270D	15.64	57.34	3.51
8	G282B	13.94	59.47	3.10
8	G273A	15.57	62.14	3.49

*^a^* Linkage group; *^b^* Coefficient of determination.

**Table 2:** Locations and genetic effects of quantitative trait loci (QTLs) detected for seed phorbol esters content in the F_2_ population of Chai Nat × M10 by inclusive composite interval mapping.

QTL Name	LG *^a^*	Marker Interval	Position (cM) *^b^*	LOD Score	Confidence Interval (cM) *^c^*	PVE (%) *^d^*	Additive Effect *^e^*	Dominant Effect	[d/a] *^f^*
*qPE3.1*	3	CN_SSR326–CN_SSR325	153	3.82	152.5–156.5	14.10	−0.09	−0.41	4.56
*qPE8.1*	8	NG288C–NG286A	50	4.06	48.5–51.5	15.49	0.31	0.02	0.06

*^a^* Linkage group; *^b^* Position on the linkage group; *^c^* 1-LOD support of the QTL; *^d^* Percentage of phenotypic variance explained by the QTL; *^e^* Additive effect of alleles from Chai Nat 10; *^f^* Dominance-to-additive effects ratio.

**Table 3:** Annotated genes located between markers CN_SSR325 and CN_SSR326 flanking *qPE3.1* and between markers NG288C and NG286A flanking QTL *qPE8.1* that controls seed phorphol esters content detected in the Jatropha F_2_ population of Chai Nat × M10 by inclusive composite interval mapping. The data are from *Jatropha curcas* Database (JCDB).

***qPE3.1***				
**Name**	**Position**	**Annotation**	**GO Term**	**KEGG**
LOC105634082	NW_012124225.1:1070722..1072343	Uncharacterized protein At4g22758	-	-
***qPE8.1***				
**Name**	**Position**	**Annotation**	**GO Term**	**KEGG**
LOC105650633	NW_012130064.1:251091..252362	Peptidyl-prolylcis-trans isomerase CYP26-2, chloroplastic	GO:0003755	K03768
LOC105650634	NW_012130064.1:244937..251195	Uncharacterized LOC105650634	GO:0008270	K03768
LOC105650635	NW_012130064.1:253456..257226	Single-stranded DNA-binding protein, mitochondrial	GO:0003697	K03111
LOC105650637	NW_012130064.1:259463..262260	Uncharacterized LOC105650637	-	-
LOC105650638	NW_012130064.1:262323..266102	Uncharacterized LOC105650638	-	-
LOC105650639	NW_012130064.1:267598..271421	Zinc finger BED domain-containing protein DAYSLEEPER	GO:0003677, GO:0046983, GO:0003676	K03680
LOC105650640	NW_012130064.1:273262..278864	Translation initiation factor eIF-2B subunit delta	GO:0044237	K03680
LOC105650641	NW_012130064.1:288075..288870	Oleosin 1-like	GO:0016021, GO:0012511	-
LOC105650642	NW_012130064.1:291937..292732	Uncharacterized LOC105650642	-	K13111
LOC105650712	NW_012130064.1:279771..287264	Pseudogene	-	-
LOC105650643	NW_012130064.1:297597..314028	Suppressor of mec-8 and unc-52 protein homolog 1	GO:0005515	K13111
LOC105650713	NW_012130064.1:317012..317392	Inactive protein FON2 SPARE1-like	-	-
LOC105650644	NW_012130064.1:324023..347807	Transcription initiation factor TFIID subunit 2	GO:0008237, GO:0008270, GO:0005488	K03128
LOC105650714	NW_012130064.1:367045..368556	Transcription factor CYCLOIDEA	-	K08735
LOC105650645	NW_012130064.1:376579..386656	DNA mismatch repair protein MSH2	GO:0005524, GO:0030983, GO:0006298	K08735
LOC105650646	NW_012130064.1:392247..402394	Uncharacterized LOC105650646	GO:0005515, GO:0008270	-

GO Term: Gene Ontology Term; KEGG: Kyoto Encyclopedia of Genes and Genomes.

## Supplementary Materials

The following are available online at www.mdpi.com/2073-4425/8/8/205/s1. Table S1: Sequences and polymorphism of 742 amplifiable primers to screen polymorphism between Jotropha cultivars Chai Nat and M10.
