# Supplementary material for: Mapping of QTLs for Seed Phorbol Esters, a Toxic Chemical in Jatropha curcas (L.)
Source: Genes (Basel). 2017 Aug 18;8(8):205. doi: 10.3390/genes8080205 (PMC5575668; doi:10.3390/genes8080205)
Supplement: Supplementary file 1 [file genes-08-00205-s001.pdf]

Supplementary File 1: Sequences and polymorphism of 742 amplifiable primers used to screen polymorphism between jatropha cultivars Chai Nat and M10

**Primer name**	**Forward primer sequence**	**Reverse primer sequence**	**Polymorphism**
MPN050	TGAAGTACCAGCCATGGAGA	TGCGTCCTTAGCGTCTTTTT	Monomorphic
MPN051	ATGGAAGCCAGTTTTTGGTG	GAGGAAGAAGCAGAGGCAGA	Monomorphic
MPN052	TCGCCTTCTGCTTCCTACAT	ATTGAGATCCGGTGGATACG	Monomorphic
MPN054	CATTCTTCCGGAACCAAAAA	TGGCGTCTATTTGCCTCTCT	Monomorphic
MPN055	TGGCGTCTATTTGCCTCTCT	CATTCTTCCGGAACCAAAAA	Monomorphic
MPN056	CGACCATAAACCCCTCTCAA	GGACGAGTCTGGTGGAGAAA	Monomorphic
MPN057	GAAGGAGGGCATCACTATCG	TAGCAGTCCTCCCAGAAAGG	Monomorphic
MPN058	CAGGTGCCCAAAACATGATA	CCCAAAGAAGGGGTAGATCA	Monomorphic
MPN069	GAAATGCAAGGATGAAAAAG	CCTGAGAAGAGGACCTTAGAA	Monomorphic
MPN071	ACAATCTTCACAAATCACCAC	ACTGATACAGCGAATTTCTGA	Monomorphic
MPN073	TCTTTCCTTTCTTTCTTTTCC	TAGACCAAATGTTAGCGAGAA	Monomorphic
MPN074	AATCTTCTTTAGCCACCCTTA	CCTCCATCTCTGTATTTTCCT	Monomorphic
MPN075	TTTGTTATCTTCTCCAAATGC	TGAGAAGAGGACCTGAGAACT	Monomorphic
MPN077	TTGGTCAATACCCATTTGATA	TAAGACCAGAAATGTTTTCCA	Monomorphic
MPN078	CGCCTTTTCTCTAACTTTCTC	CTGATTTGATGAGAAGCAAAA	Polymorphic
MPN080	AAAAGAGAGTTAGAGCGAACG	CTTCTCCTTTTCCATTAGAGG	Monomorphic
MPN081	CCTCTTTCCCTCTAGTTCTGA	CACTACTAACACCGCCAATAG	Monomorphic
MPN082	AGAAAGAACCTCTGTCCAATC	AGAAGAGGACCAGATCAAATC	Monomorphic
MPN083	GATTTTGCTGTTGGAGTTTC	TCATCATATCTTCTCCCTCAC	Monomorphic

MPN084	GAGCAGCAGTGTTGAAGTAAC	CCCTCTATGGAAAACTGAGAT	Monomorphic
MPN086	GAAAGTCAGTTGAAAAGGTTG	TTCTTATCTGCTCTTGCATGT	Monomorphic
MPN088	CTTAGAATTTCCCTTCGAATC	TCTATCGAGCTTTCGAAGTAG	Monomorphic
MPN090	CTTAGAATTTCCCTTCGAATC	TCTATCGAGCTTTCGAAGTAG	Monomorphic
MPN091	AAGGAGATCCTGATCTGAGAA	TTGGGTAAAGTGCAACTAAAA	Polymorphic
MPN092	AAAGTTTGGTACAAGTGCAGA	GGAGTGATACTTGTTCTGCTG	Monomorphic
MPN096	CGATCCAAACGTTACAAATAA	GAGAGAGAGAAGAGAGGGAGA	Monomorphic
MPN097	TCTGTTTTGGTGAAGTCAGAT	AAGCCATTGTTTCTACTCACA	Monomorphic
MPN099	CATGTAAATGTCTCTTCTTGGA	AAGCAAAAATAACGGATTCAC	Monomorphic
MPN101	TAACCATTTGATTTGATGGAG	CCAACTCATGGCTTAATTTTT	Monomorphic
MPN103	AAAAGTAGTATCGGTGCTTCC	CTTCAGATATGGAGATGCAAG	Monomorphic
MPN104	TAACCATTTGATTTGATGGAG	CCAACTCAATGGCTAATTTTT	Monomorphic
MPN105	TTCATGGACTAATCAACTGCT	AACATAAGCAACTCCCCTATT	Monomorphic
MPN106	TTCACAGTTCCAATTTTCCTA	TGAGTTTGGAGAGAGTTGAGA	Monomorphic
MPN108	GCTCAGACAACAAGAAATCAC	ACTAAAAGAGAAAGCGGTAAT	Monomorphic
MPN111	CTCGCAGAGATGAACTCTAAA	AAAAGAGATGGCAAAGAAATC	Monomorphic
MPN112	AGAAAGAAACAAAGGAAGTGG	GTCAGTGGTAATTGATGTTGG	Monomorphic
MPN113	TTGAGCTGCAAAATAAGGTAA	AACCTACCAAGTCATGTTTCA	Monomorphic
MPN115	CACCCTACTAAGCAGTTCAAA	TGAGCTAAAATAAGTGGGAAA	Monomorphic
MPN116	AGAAATACACCAGACAAGCAA	AGTTTGGACAGCATGTAACAG	Monomorphic
MPN117	TCGAGAACCAAAAACATTTAC	TACTCATTACACTTCCCGTTC	Monomorphic
MPN118	ATAGGAACTTCAAAGCGTTTC	TAAGCATAAACACGCACTATG	Monomorphic
MPN119	ATGCCTTTTGAATGGTAGAAT	ATCAAAAAGCTTTAAGGGACA	Polymorphic

MPN120	AAAATAAAAGAGTGGGCTTTC	TAAACTGCCTTGTGAAATCAT	Monomorphic
MPN121	TCACATCTGACATCTATCCTTC	AAAATGGAGAGATCAGAAACC	Monomorphic
MPN122	AAAATAAAAGAGTGGGCTTTC	TAAACTGCCTTGTGAAATCAT	Monomorphic
MPN123	TTTACCGTCAACTCTCTTGTG	ACAGACACCACAACCAGTAAC	Monomorphic
MPN124	TTCACTTCTGGTCTTTGCTTA	CAGTGATTTTGTCTCCATCAT	Monomorphic
MPN125	GGAGGCTCTATAGCTCTTGTC	ATGTGTTCCATCTTCTTCTGA	Monomorphic
MPN126	AGGAATAAAGAAACCTAGCACA	TCTTCTACTCCTCCCTCTTTC	Monomorphic
MPN127	CAAATTAAATTCCAGCAACAG	ATTCCAAACAAACTTCTCCAC	Monomorphic
MPN130	CGATAGCTGCATTAAAAATTG	GAATTGCGATCACAGTAAGTC	Polymorphic
MPN131	TACAATGATGGTAACGAGGAG	TATAGATCGAAGGGGTCTTTC	Monomorphic
MPN132	AGGAATAAAGAAACCTAGCACA	TCTTCTACTCCTCCCTCTTTC	Monomorphic
MPN134	TTCAGCTTTATTTCTCATCCA	CCTATGGTGTCTCCGTATGTA	Polymorphic
MPN135	CGATTTCTAGAGAGAGAAGCA	ACCTTTGAAAGTACCCAAAAC	Monomorphic
MPN136	GGAAGATCTAAGAACCCTGAA	AGTCTCTTCCGTTTCAAATTC	Monomorphic
MPN137	CGATTTCTAGAGAGAGAAGCA	ACCTTTGAAAGTACCCAAAAC	Monomorphic
MPN138	TCACTCTCTCCCTCTCTCTTC	ATCAGATCGGCTTCTCTATCT	Monomorphic
MPN140	AAGGAAAAGCGAAGAAAGATA	GGAGTCTTGAATCTCATCCTC	Monomorphic
MPN142	AAAGGAAACCGTAAGTTTGAG	CCTTTGTCTTCTTTTCTTTCC	Monomorphic
MPN144	TTCTCTCGGAACAATCTACAA	TGCAAAACAGGAGGATTAGTA	Monomorphic
MPN145	GCCCATATCATTTCTCTGTTT	ATGAGTCACACTCTTGGTTTG	Monomorphic
MPN146	AACAAATCTGCAACAAACCT	CGACCCTTTTACATTACACAG	Monomorphic
MPN147	TATCCTGTATTGGGCAGTTAG	AGGAGACACAAGGAGATTTTT	Monomorphic
MPN148	AACCCGGCACTCTAGTTATAC	GAAGTGAGCAGAAACAGAAGA	Monomorphic

MPN149	GAGGAACTAACCACTCCATTC	ATCCGGGAAAATAAATCAAA	Monomorphic
MPN150	CCCCAAATAATTCAATTTCA	CTGTGCCCATTTTTAGAGATT	Polymorphic
MPN151	CTTAAAAATCGGGTCTTTTTC	CAGAAAAGGGATCTTCCTAGA	Monomorphic
MPN152	GTTTGTGTGTGCTGTCTACTTT	TTCTTCTCTTCTCCCATTTTT	Monomorphic
MPN153	TGAAAGCACAGTGACATTAAA	ATCGAAGAGTGAGAAATGGAT	Monomorphic
MPN154	GGACGTGAATATAGGTTCCAT	TGGATGAATGGTTAAATCTGA	Monomorphic
MPN155	CTCCAGGACCAAAAGTAGATT	TGAAGCCAGTTAAAGGATAGTT	Monomorphic
MPN157	ATTGAAACTGTTGTGGACACT	GGAGATAAGGAAAAGGATTGA	Monomorphic
MPN159	AAGCCATTGTTTCTACTCACA	TCTGTTTTGGTGAAGTCAGAT	Monomorphic
MPN161	ACAAACGCACTGACACTAAAT	AGTCGAAGAGGAAATGAAAAG	Monomorphic
MPN162	CATTAACGGTCATTTCATTTC	TTTCAGTTTCAAATGGTCACT	Monomorphic
MPN163	CCTCTTTCCCTCTAGTTCTGA	CACTACTAACACCGCCAATAG	Monomorphic
MPN164	CAACCCATTATCCCACTAAAC	AAACTTCTGAAGAAACCCAAC	Monomorphic
MPN165	CTGTTTGTCATCAATACAGCA	TTCGTCGTTAGAGAAGTCAAG	Monomorphic
MPN167	ACCTATATTGCATGGTCATTG	GAAGGGAAGAGGTTTATATGC	Monomorphic
MPN170	CTAGGTCCGGTTCTTAATGAT	TTTCAATTCAGTGAAGTTTCC	Monomorphic
MPN173	AAAAGCTCTTTCTTTCCCTTT	GTTGACATTGGAGGAGAGAA	Monomorphic
MPN174	GGTGCTATAATGATGGTAACG	TATAGATCGAAGGGGTCTTTC	Monomorphic
MPN175	TACAATGATGGTAACGAGGAG	TATAGATCGAAGGGGTCTTTC	Monomorphic
MPN181	TATGATTTCTTCTTCCCCTTT	GAGGTTGGGTTGCTAAATTAC	Monomorphic
MPN183	TACAATGATGGTAACGAGGAG	ACGTATAGATCGAAGGGTTCT	Monomorphic
MPN185	AAGGAAAAGCGAAGAAAGATA	GGAGTCTTGAATCTCATCCTC	Monomorphic
MPN186	TCTCCAGTTCTACCAGGAAGT	CTTATTTTGGCTGTTTGTTGT	Monomorphic

MPN188	AATATCAAACCCACCAAGAAC	AGCAACCTTACCCTTGTCTAC	Monomorphic
MPN189	TTAAACATAACCCTCCTCCTC	GTTGTTGTAGCCTTAGCAGAA	Monomorphic
MPN192	GATCGCTTACTTTAACGGTTT	TTGTTGATGGGTTTCTTCTTA	Monomorphic
MPN193	CCACTGTTTCTTTCACTCTGT	TCCCTCACTCTCACATGTATC	Monomorphic
MPN195	ATCGGATTATGGGTTTTCTAC	TGTTTTAAATTCATCCTCACG	Monomorphic
MPN196	CCACTGTTTCTTTCACTCTGT	TCCCTCACTCTCACATGTATC	Monomorphic
MPN197	GCAGGAGGTAGTTAAGAACAA	TAACAGCTCTTAATCCATTGC	Monomorphic
MPN199	CCTCAACCGATATTACCTTCT	TCACTAAAAGGATTCACCTCA	Monomorphic
MPN200	AATCAAAACAATCCCCATTT	TTCTGTATCATTCCGATGTTC	Monomorphic
MPN202	TCATCAACAGAAAACCAATCT	GCAACAACGAGAAAGATTACA	Monomorphic
MPN204	ACTCATAGGCAATTGTTTGAA	CAACGTACTCAGACAATAATGC	Monomorphic
MPN205	AGCTAAGTTAATCAGCGGATAC	ATGTGGCTCTGATAGAGTCTG	Monomorphic
MPN206	TGTTCACTCACAATTGTTTCA	GCAGATTCTTGATGATTGATT	Monomorphic
MPN207	GCTTTTACCCTTCTTTTGAGT	GAGTTCTGTGAAGAAGAGTCG	Monomorphic
MPN212	TGAACCACCAAGAAAGAGATA	GATTTATCAAAGCCCTCTAGC	Monomorphic
MPN223	TTCAAGAATCCAATACCACTG	GATATCATAGAAACGCCTTCA	Polymorphic
MPN226	CACCAACAAAAGGAATTAGAA	GGGAAAGGAAACAGTAAACTT	Monomorphic
MPN227	CACCTTTGTTTTCCTCACTAA	TTAATATCCGATGATGAGACG	Polymorphic
MPN228	TGGTGATTCTGAAGTTGATTT	TCCCCAACACAAGAATAATAA	Monomorphic
MPN231	CGAATTGCAAACTATATCTCC	GAATCTAATGCCCTGACAAG	Monomorphic
MPN232	TGGTGATTCTGAAGTTGATTT	TCCCCAACACAAGAATAATAA	Monomorphic
MPN233	CTTGGAGACTGGTTGTTAGTG	CAAAGATTCCAGTTGAAGTTG	Monomorphic
MPN234	CGCCTAAAAGAAAGCTCTAAA	TCACAAGAGAGATTGTTTTGG	Monomorphic

MPN235	CTCCTTGCTGATTTCTTTCTT	TATACAACGCACAAACATGAC	Monomorphic
MPN236	AGCAAATTGAGGTTCTTTTTC	ATGATTTGCAGTTACGATTTG	Monomorphic
MPN237	AAATGAATATGTACAGCAGCAG	CTTTAATGTCAGTCGAAGTGC	Monomorphic
MPN238	ACCAAAATGTCCTAATCACCT	GGTTGGACAGAGTATGAGAATC	Polymorphic
MPN239	CGTCTCTAGATTCCCTGAAAC	GAAATTTGAGAGACCCAATTC	Monomorphic
MPN241	TCAGATTTCTATGCATTCCTC	CTGTGTCACATATCCCAAAGT	Monomorphic
MPN242	CGATTCTCTCACTCTCAAATG	ATAATGCGAAAGGGGTATTAG	Monomorphic
MPN245	GCATCTTTCTCTTTCTCCCTA	ATGAGCGAAGTCATGGTAATA	Monomorphic
MPN247	TTACCTCCATCAACAACAATC	TGTTGAATTAAACGCTGTTG	Monomorphic
MPN248	GGCCTTCTCAATCTTTACTTC	CACAAGTGCTTCTCTCTTTGT	Polymorphic
MPN249	CAGGAAATTGAGTTTCAACAG	GAATAAAATCAAGCAGGGAAT	Monomorphic
MPN250	CGTCTCTAGATTCCCTGAAAC	GAAATTTGAGAGACCCAATTC	Monomorphic
MPN252	CCATCTCTCTCATGTTCTTTG	GAACTACATGCAGGAAGGAA	Polymorphic
MPN253	AAAAACTGAATATGAGGAATCG	CCTTAAAACATGCCAAAAGTA	Monomorphic
MPN254	ACATTATTAATCTTCTCGTGCAT	AGAAAGGAAATGCTGAATAGG	Monomorphic
MPN256	CTACTCTGAACCTTTCCCATT	TCTTCTATAACCAATGGCTTG	Monomorphic
MPN257	ATCTCGCATATGTAGTGTGGT	TTGCTAAATTACACTGGAGGA	Monomorphic
MPN259	CAGCTTTATTTCTCATCCAGA	CCTATGGTGTCTCCGTATGTA	Monomorphic
MPN260	CAGCTTTATTTCTCATCCAGA	CCTATGGTGTCTCCGTATGTA	Polymorphic
MPN261	CATTTCAAGCTCAGTTTCTCT	TAAAACCCTTGTGTCTTTCAC	Monomorphic
MPN263	CTTCTATATACCGACGAGCAA	ATCCATGATGAGATCAAACTT	Monomorphic
MPN266	GCTCTTTTGCTCAATTCTCTT	GCTTCAAAAGTAAGCCAATAGT	Monomorphic
MPN268	ATCCTGTGCCTTACTGTTCTA	CATGTGGATAAATGGAAAAAG	Polymorphic

MPN269	GTCAAAACTTCCTCTGGTTTT	ACTGAAGACATTTTTGCCTTT	Monomorphic
MPN273	CAGCTTTATTTCTCATCCAGA	CCTATGGTGTCTCCGTATGTA	Polymorphic
MPN275	ATCAATAGTGCCCTTTCTCTC	GAAACACTCTCCCTCTCTCTC	Polymorphic
MPN280	CGGAAAAAGAAAGAGAGAGTT	TTCCTTTCTAGTTCAATGTCG	Polymorphic
MPN286	GAAATTCTTCCTGAGTGTCCT	TGCAAGACAGGAAATTGATAA	Polymorphic
MPN288	CTGTCACTGGTAGACAGACAC	AAAATGCTTCTGAGAGAATGA	Polymorphic
MPN291	CCATAGCAAAGTTGTCTTGTG	CAAGTTTACCAGCTCACAATC	Polymorphic
MPN293	AACCGCAACAAGAAACTGT	ATTTCCATACGTCTTCCTCAT	Polymorphic
MPN295	TTGCTAGATTGCTAGCTTGTC	GAAGAAAGAGGAAATGGGTAA	Polymorphic
MPN296	TCCAAACAATTGGATAGTGTC	CATTGCAAGGAGTGTAAGTAAA	Monomorphic
MPN297	TCAAGTGAGACTGTAGTGGAGA	CCTGAGAGACACAGCTGATAG	Monomorphic
MPN299	TCATATTACGAACCCTTGAAA	TCCTCGTATAAATGAACCAAA	Monomorphic
MPN300	CTCCTTGCTGATTTCTTTCTT	TATACAACGCACAAACATGAC	Polymorphic
MPN301	AAATTTCCTTTGTGAGCTTCT	TACACCCACTTGACATCTCTC	Polymorphic
MPN302	TGAGACTGTAGTGGAGAAGAAA	CCTGAGAGACACAGCTGATAG	Monomorphic
MPN303	CAGAGATTGTGAATTGCATTA	CCATGAAAATCGTGTAGTCTC	Polymorphic
MPN304	TAATCCGAAAACAAGAGTGTG	CTGTTCTTCCTCTTCGATTCT	Polymorphic
MPN305	CTCCACACAAAAACTGAGAGA	TGATTAACAGAAAGGGATTCA	Polymorphic
MPN307	ATCTCTCTCTCCTTCACCATC	CAACTTCAACTTCAGATCGAC	Polymorphic
MPN308	CCACACAAAAACTGAGAGAAA	CGAAAGACCAGATGATAACAG	Monomorphic
MPN309	CATTCACATGAGAGTTAAGTGC	TGCACAAGTCAACAGCTATC	Polymorphic
MPN313	CAGTGGTATCAAGGCAGAGTA	ATATTCGTTGCTCATAATGGA	Monomorphic
MPN314	GTGACCTCATCATAGCAGAAC	GCAAATCAGAAGCTCAACTC	Monomorphic

MPN321	CAAACGGTATTGTCGTGTATT	TGTTGTAGTGGGTGGGTATAA	Monomorphic
MPN323	AAGAGAAAGGCAACTCACC	ACCGATCTCTTAGCTCAAAAC	Monomorphic
MPN328	CACACTTCAGCTTAATTTACACA	AAAAATGTTGATTGTTGTCG	Monomorphic
MPN329	ACACCTCCTTCTTCTTCCAC	TTCAGAAAAGAGTTGAGCTTG	Monomorphic
MPN331	ATTCATCAACCACACTTGAAC	AACAGAGAAATATGCGAGTGA	Polymorphic
MPN332	ATGTCGATGGGAATATCAAAT	TGAAGTTTTTGAATCTTGGTC	Polymorphic
MPN333	CAAGTTTCTGAGGATAAAGAGG	CTCTTGTTGCTTTTGTCAGAG	Polymorphic
MPN334	CTTATCCTTCCCAACTGTGAT	AAAGGATGAGTAACACAGACAA	Monomorphic
MPN335	GCAAAGTTTAGCATTCAATCA	CATTATGGAACATGAATGGTG	Polymorphic
MPN336	CAGGAACATCTAGCTGAAGG	CTAATTAGCAACCGTCAAAAA	Monomorphic
MPN341	CATGACACACCACTACCCTAT	CAGGAAACACAATCAAGTCTC	Monomorphic
MPN343	TTCTTCTTCCTTTCCAGATTC	GCCAAAAGTATAATCACTCCA	Monomorphic
MPN344	TTCACGAGCTTTTGGTCTAT	CATACTGGTCCCAACTACAAG	Monomorphic
MPN345	ATGTTAGGAAATTGGAAAGGA	TGGGTTATTCTTGTGTATGCT	Monomorphic
MPN346	CAAAAGTGGGTTTTAGCTC	GCAAAGAAGAACTTGATTGAT	Monomorphic
MPN347	TGGTTCTTGTTTACTTTCCAG	GTCACAGGAGAGGAGTAGCTT	Monomorphic
MPN351	ATCTCTCTCTCCTTCACCATC	CAACTTCAACTTCAGATCGAC	Monomorphic
MPN352	CAGACTGCTTAGTTAGACTTGT	CTATGAAGAAGTGTCCGAATG	Monomorphic
MPN355	TGATTGGTGCTTTCTTTATGT	CGTGTGCAGAATTAGCAGTAT	Monomorphic
MPN359	CAAGTTCTTTTCCACAACTCT	TTCTCTCTGATGAGATGGTTC	Monomorphic
MPN362	CTCAAAGCCAATAAGCCTAA	GTCCTTAGCTGAGAATGATCC	Monomorphic
MPN363	CAAAGCCAATAGGCCTAAAC	GTCCTTAGCTGAGAATGATCC	Monomorphic
MPN364	ACATTACATACATGTGAAAGAGG	CCATTCCCTTAATTTGATATG	Monomorphic

MPN365	AAACTTTCAATCTCCAATTCTC	GTGAGTTATTGCGAAAGAAGA	Monomorphic
MPN366	AAAGAAAACACAGATACGAACA	GAATTACTCCCTCGTGAAAAC	Monomorphic
MPN367	GAAAGGCAACTCACCAAAC	ACCGATCTCTTAGCTCAAAAC	Monomorphic
MPN369	AACAGAGAAATATGCGAGTGA	ATTCATCAACCACACTTGAAC	Polymorphic
MPN371	GCATCTTCACTTCAGTATTCA	AATTTTTATCGATGGAGATGG	Polymorphic
MPN375	AGCGTTTCTTTAAGCTGTATTC	AATCAACAAGAGGTAGTGGAGT	Monomorphic
MPN376	GAAAGGCAACTCACCAAAC	ACCGATCTCTTAGCTCAAAAC	Monomorphic
MPN377	GAAAGGCAACTCACCAAAC	ACCGATCTCTTAGCTCAAAAC	Monomorphic
MPN378	AACAAAAGAGAAAGGCAACTC	ACCGATCTCTTAGCTCAAAAC	Monomorphic
MPN379	AACAAAAGAGAAAGGCAACTC	ACCGATCTCTTAGCTCAAAAC	Monomorphic
MPN380	GAAAGGCAACTCACCAAAC	ACCGATCTCTTAGCTCAAAAC	Monomorphic
MPN381	GAATTCATGTCTGATTTTCCA	CAAACAAACCCTAGAACTGAG	Monomorphic
MPN382	CAAAGCCAATAGGCCTAAAC	GTCCTTAGCTGAGAATGATCC	Monomorphic
MPN384	GCTTCTTCTGTTTTGTCCATA	ATGGTTGGTGAGTGCTAAGTA	Polymorphic
MPN385	AACAAAAGAGAAAGGCAACTC	ACCGATCTCTTAGCTCAAAAC	Monomorphic
MPN386	GAAAGGCAACTCACCAAAC	ACCGATCTCTTAGCTCAAAAC	Monomorphic
MPN387	GCTTCTTCTGTTTTGTCCATA	ATGGTTGGTGAGTGCTAAGTA	Polymorphic
MPN391	GCTTCTTCTGTTTTGTCCATA	ATGGTTGGTGAGTGCTAAGTA	Polymorphic
MPN392	GAAAGGCAACTCACCAAAC	ACCGATCTCTTAGCTCAAAAC	Monomorphic
MPN393	GAAAGGCAACTCACCAAAC	ACCGATCTCTTAGCTCAAAAC	Monomorphic
MPN395	GAAAGGCAACTCACCAAAC	ACCGATCTCTTAGCTCAAAAC	Monomorphic
MPN396	GGACTCTTCTTCTTCTTCGTC	GATGTTTTCTCCTGCAATACA	Monomorphic
MPN398	CTCGATCCTCCTTTCTACTTC	GATTGACCACAAAAGTGAGAC	Polymorphic

MPN399	AACCGCAACAAGAAACTGT	ATTTCCATACGTCTTCCTCAT	Polymorphic
MPN400	TTAAAATCATGCATGTCACCT	GACTTCCCCTATGAACCTAGA	Polymorphic
MPN401	GAAAGAGCAACTCACCAAAC	ACCGATCTCTTAGCTCAAAAC	Monomorphic
MPN402	GATTACATACATGTGAAAGAGG	CCATTCCCTTAATTTGATATG	Monomorphic
MPN403	GCTTCTTCTGTTTTGTCCATA	ATGGTTGGTGAGTGCTAAGTA	Polymorphic
MPN404	AACAAAAGAGAAAGGCAACTC	ACCGATCTCTTAGCTCAAAAC	Monomorphic
MPN407	GAAAGGCAACTCACCAAAC	ACCGATCTCTTAGCTCAAAAC	Monomorphic
MPN410	CCACAGATTAACCCAAAACTA	AGTGTTTAGTGAGCAGCAGAG	Monomorphic
MPN413	ATTTTGAATTGGGAACTGAA	CCACCCTCTCTATTTCTCCTA	Polymorphic
MPN414	GAAAGGCAACTCACCAAAC	ACCGATCTCTTAGCTCAAAAC	Monomorphic
MPN416	CAGAATACCGTATCAGAATATCA	GAGACTCCAGAAGGAAGGTAG	Monomorphic
MPN417	ATAACAAAAGAGAAGGGCAAC	ACCGATCTCTTAGCTCAAAAC	Monomorphic
MPN418	TTGCTAGATTGCTAGCTTGTC	GAAGAAAGAGGAAATGGGTAA	Polymorphic
MPN420	GTCTCTGCTTCTGTGACAAAC	GAGGAGTGAGGGAAAGAGTAA	Monomorphic
MPN422	GGATCAGAGACAAATACTGAT	ATGGATATTAGCGGTGGTTAT	Polymorphic
MPN423	GGGAATCACTCAAAGCCTAT	ATTTGAATCCAAGGAGAAAAG	Monomorphic
MPN424	TAAGCAAATTTACCTTCCACA	CAGTATGATGACACCATCTCC	Monomorphic
MPN425	CTCCTCTCGTTTCATTTCTCT	GGAAAGAGACACGCTAGATTT	Polymorphic
MPN426	GCAGTGGTTATCAGAGACAAA	ATGGATATTAGCGGTGGTTAT	Polymorphic
MPN427	AAGTGTGTGTCCCTGTGTG	AGCGTTTAAGAGAAGGAGGTA	Polymorphic
MPN428	GTTTCTTCTACAACTGCAAGC	GCAAAAGAAACGTAGTAAAACA	Monomorphic
MPN429	CTCCACACAAAAACTGAGAGA	TGATTAACAGAAAGGGATTCA	Polymorphic
MPN430	TATCTCTTCGCAATCACTTTC	GGTTCTTCTTTACCCATTGAT	Monomorphic

MPN432	GTGAGATCCCCTAATTCTGTC	CAAGAATTAAAAGAGGGGAAA	Polymorphic
MPN434	ATCACAGCGCATACCAAA	AAACCTCAAAAGAACAACGAT	Monomorphic
MPN435	CGAGCGTCTCCTACCTCT	TATGAAGAAAAGGAAATCTGC	Polymorphic
MPN437	GGGAAGGTTGGCAGTCTAT	GGTTCTTTGAGCTGGTAAGTT	Monomorphic
MPN438	GGTCTGTCATTGGGTATTGTA	AGTTTCTCATCTTCCTCTGGT	Monomorphic
MPN439	AAGAGAGAAAGCGCAAGAG	AATCCTGGCAGTAGCTCTAAC	Monomorphic
MPN440	GCCAACCATATTATCCTTTTT	CATGAAGATTGAAAGAAGCAG	Polymorphic
MPN441	ACCATAGTAGCCCAGTAGTCC	TAAACAAGGATCAGATCAAGC	Monomorphic
MPN444	ACCATAGTAGCCCAGTAGTCC	TAAACAAGGATCAGATCAAGC	Monomorphic
MPN445	TTTTCTTCTTTGCTTCTTCTG	GAACTAATATGCATCTCCAATG	Polymorphic
MPN446	GGGTCATCAAATGAAGTGTAA	GAAGGAGGCATAGCTACAGAT	Monomorphic
MPN447	AAAGTTTCACCTTCTCTTTCC	GACCTCTAAACAACAATCCAA	Monomorphic
MPN448	AAGAGTCCTCCTTTTGGTAAA	GACAAAGAAAGAGAGAGCACA	Monomorphic
MPN450	GAATTCATGTCTGATTTTCCA	CAAACAAACCCTAGAACTGAG	Monomorphic
MPN451	GTAAAGAGGAACGCAGAATG	TCAAAACTTGAAGGATGAAAC	Monomorphic
MPN452	CAAATAACCTTTTCAAGTCCA	GGTGAAGAAATGTTTTCAATG	Polymorphic
MPN455	AAACTGCAGATTTTTATTGAGAG	GCATGCCATAAGAAATACAAA	Monomorphic
MPN457	CGTTGTCTGGGACATAGTG	TTTTGAAACAAGAAGTGGTTC	Monomorphic
MPN459	AGACAAAGGAGGTGAATTCTT	CAGCATCCATAAATTCAGAAG	Monomorphic
MPN461	AACTGACACAAAGATCCAATC	ACAGGAGAATCGAGACATTTT	Polymorphic
MPN462	TCCATCTTTGAAGTCTCACAC	GTAAAAGGCAATTTAGGTGGT	Monomorphic
MPN463	CCACAGATTAACCCAAAACTA	AGTGTTTAGTGAGCAGCAGAG	Polymorphic
MPN464	GATCCAAACATTGCCTTTC	TCCAAATTGCAGATAAGAGTT	Monomorphic

MPN465	AGAAACAATATTTCCCTCTGC	TCATATTCGTTGCTCATGATT	Polymorphic
MPN466	TCTCTATATTTGCAGGGGAAT	CTCTGACATGGAGAGAAACAG	Monomorphic
MPN467	CGTTAGTTAATGTCCCTGAGA	GAGGGAGAAGAATTGAGAGAA	Polymorphic
MPN468	CTTACAAGCCATTAAGTGACG	CACAACATTTCTTCTCCACTC	Polymorphic
MPN469	TCTGTTTCTTACTTCTCTTTTCC	CCTTTGTTTGTGTGTTCTTTC	Monomorphic
MPN470	CACATACATAGGGCTTTGTTC	AAATGGTGTGTTTGGTTACAG	Monomorphic
MPN472	CTCTTAAACGCTCTCATTCAG	CAATATCATCAGCCATCATTT	Monomorphic
MPN474	GATCAAAGATCACACCAACAG	AGCAGAGAGAAAGAAATGGAT	Polymorphic
MPN477	AAGCTTGAGAGTTGAAACGAT	AGCAGAGAGAAAGAAATGGAT	Polymorphic
MPN478	CAGCTACTGATGATGGACTTT	GCCTTCTTGCTTTCTGTTTAT	Polymorphic
MPN481	TAGTAGCGCTAATCGAGACTG	CCCTTGGTCTCCTTTTTC	Monomorphic
MPN482	CAAGCCAAAGTAGCAGAAGTA	CAGAAGCAGATCAAACAAACT	Polymorphic
MPN483	AGAACAATATCATGCTCTGGA	AGGCCTAAAATAGAAACCAAA	Monomorphic
MPN484	CGCTCTTTCACTCTCTTTGA	TTATATTCCGAAAAGGAAAGG	Monomorphic
MPN485	CAAGCCAAAGTAGCAGAAGTA	CAGAAGCAGATCAAACAAACT	Polymorphic
MPN486	GACTGATTGTCAAAGCAAAAT	CAATGGTTTTCTTGGTAGTGT	Polymorphic
MPN487	GCTAATGGTGACACTGATGTT	GCCCTTCCAACTGTACATAAT	Polymorphic
MPN490	GCTTGCTGTGATACTACTGCT	AATCAACACCTAACCTTATCTGA	Monomorphic
MPN491	AAGAGAGAAAGCGCAAGAG	GAATCCTGGTGGTAGCTCTAA	Monomorphic
M10_SSR4	ACCTTCCTGAGTCGGTGAGA	AATAGGCTGGGGAAGATGCT	Monomorphic
M10_SSR7	TCTCAGGGAAAAACCCTTCA	GCAGACACTTTGGTGCTCAA	Monomorphic
M10_SSR10	TGAAAAAGCAAAGATCCATACA	GAAGAAACGCTTGCTTCACC	Monomorphic
M10_SSR12	CCATTGCTGCGATCCTTAAT	GGGACATACCAAAACCGTTG	Polymorphic

M10_SSR13	ATAGAACCAACGCCAGTCCA	AGCCACAGCCAGATGCTAGT	Polymorphic
M10_SSR14	TGCACAGGAACATTGAAGTAAAA	GTGAAGGGCTTGGCTTGTTA	Polymorphic
M10_SSR15	GAAGTGGGTCTCCTTCAACG	TGCTCATCATCAAGCAATCC	Monomorphic
M10_SSR18	GAAACAACAGCCTGCAGTGA	ACATGAATCCAATGCAACGA	Monomorphic
M10_SSR20	TGACATTGAATTTGGAGAGGG	CATGCTTCCAACAAGAACCA	Monomorphic
M10_SSR25	TGAGGTGCAAAATGATCAGC	AGTAGAAGCCGCAGCAGAAG	Monomorphic
M10_SSR27	GGCTCGCAGAAGAAAGACAC	AGGCTCAAACGCTAAGCAAG	Monomorphic
M10_SSR41	TGGCATGGTTGAAACAAAAA	GAATGCTGTTTCTGGGCATT	Monomorphic
M10_SSR46	CCATTTCCTTCCCCAAGTTT	GCCACTGTTGAAGAGGAGGA	Monomorphic
M10_SSR48	GCATCTTCCTCACCATCACC	CGAGTTGCCTGAACACATGA	Monomorphic
M10_SSR50	TTGCCTCTTTTCTTGGAAGC	AGGCCTAGAGAGATGGGGAG	Monomorphic
M10_SSR62	GAATTGGGAGCTCAGTCTCG	TGATATCATCATGAGCGGGA	Monomorphic
M10_SSR68	TGCAAACCTTGCTTCATCAG	GCTTAATGGCAGAGACTGGC	Polymorphic
M10_SSR71	CATATCGCTATTCCCGAAGC	CTCGAAACCTCTTGAGGACG	Monomorphic
M10_SSR75	GCCCTTTACAATTTTCTACTGTCTC	ATTGTCTTGCCATCCAGCTC	Monomorphic
M10_SSR79	TCCTCTTCCTTGTTCTGGCT	AGTTGAAAGGCAACCCTCCT	Polymorphic
M10_SSR83	CATGGTTGCTTGTCACTTGC	CGGAAAACCACTTCAAGGAA	Monomorphic
M10_SSR84	ATTGATGGCAGTAACCAGGC	AACTCCACCATCCTATCACCA	Monomorphic
M10_SSR87	GTGCTAGCAACCACAACAGC	CCACATGACCAACTTCGCTA	Polymorphic
M10_SSR93	GGACTCCAGCCAAAATGGTA	CCAACAGGCGTTTTCACTTT	Polymorphic
M10_SSR94	CAGCTCATTCAACTCGTCCA	CTGAAGAGAAGGGCAATGCT	Monomorphic
M10_SSR99	TCCGACTCCGATTTCTTCTG	CTACGGTTTCAGAGCGAAGC	Polymorphic
M10_SSR107	TCTTGTGGTGAAATGGGTCA	CTAGCTTAGCGACCGCTGTT	Monomorphic

M10_SSR110	TCATCATCAAGCTCGCTCAC	CTGATCCTGATGACGCAGAA	Monomorphic
M10_SSR112	CACTTGCAAGACTTGACAGAAAC	TGGCCTAGTCATTCCCTGTC	Polymorphic
M10_SSR125	GCTCTTCAATGGGCTCTTCA	ATGTACGCAAGCAAGCACAG	Monomorphic
M10_SSR128	TGAGGTGCATGAAATTGAGG	CCATCATCCTCTCCTTGCTC	Monomorphic
M10_SSR131	TCCAAGAGAGAATCATCGCC	TCCCGTGATTCTTGGTTTTC	Monomorphic
M10_SSR132	ATGCCACTGGTGCTAGCTTT	CCAGCACTTCTTCTTCCTCC	Monomorphic
M10_SSR135	TGCACCACTTACAGAGCTGG	GGAGAGATAGCCGGAGGAAG	Monomorphic
M10_SSR136	CAATTCCCAGCAAACACTCA	CGTTCTCAGCTGCAAACATT	Monomorphic
M10_SSR138	TCTGAGGAGAACTGGGAGGA	TGGCAAGCAGAGAGGAAAGT	Monomorphic
M10_SSR147	AGGAGGAACCGCGTTAAAAT	CACCATTCATCTTCGCACAC	Polymorphic
M10_SSR149	TGGTTATGGTTGGAAGGCAT	CACTTGCAAGACTTGACAGAAA	Polymorphic
M10_SSR150	ATGAGTTGGTTCGCCTTCAC	CCTCCATGCTTTGAGTTGGT	Monomorphic
M10_SSR159	CAATGACCTCGAGCTAAGCC	GGAAATTTGCTACTGATAACCACA	Monomorphic
M10_SSR160	GCAGTTACACGCAGCACATT	AACTCAGCTCGGCCAAAAAT	Monomorphic
M10_SSR163	GTGCCTGAAGTAAGGGCAAG	GTGAGAGGCACAGTTGCAGA	Polymorphic
M10_SSR169	TGCACCACTTACAGAGCTGG	CCGATAAGACCATCGGAGAG	Monomorphic
M10_SSR174	GTTGCTGTTGATGTTGACGG	GGTATTGCGGTGGATAATGG	Polymorphic
M10_SSR181	TGTCTCAACTCGAGAACCCA	GCAGCTGTTAAACCAGCCAT	Polymorphic
M10_SSR183	TTGCCGGAGAATGCTAAGAT	TCCACATACCACAAAACCCA	Polymorphic
M10_SSR185	CATCGGTGGTGGTATTTTCC	CACAGCTGATAGGGATGAACAG	Monomorphic
M10_SSR209	TTCCGATCCCTCAAACAAAC	ACCATGCATCACCATGACAC	Monomorphic
M10_SSR217	AGCTGGAGAGGAAGGGCTAC	TGCATGTCTGCACATTAGAGG	Monomorphic
M10_SSR221	TGAAACAGCCTGCCTTATGA	TCTTCCGCTGCTGACAAAAT	Monomorphic

M10_SSR222	GGCCACTTGTATGGGACTTG	AGCTCCAACTACCCTCCGTT	Monomorphic
M10_SSR225	CAACAATGGCATGCTCAACT	AAGGCATTACCAGTTGGTCG	Monomorphic
M10_SSR230	AGGGGCACCTTTCAAAAACT	TGTGGGTCGTGGCTGTAGTA	Monomorphic
M10_SSR249	TCTACCCAGCAACAAAAGCA	GCACCACTTTCACTGCAGAA	Monomorphic
M10_SSR256	CACTTCCCTCTCAGGCAGAC	TCGAGCTCATCAGGATCGTA	Monomorphic
M10_SSR260	CAATTGCTGCCATTACATCG	CCAACTCTTTCTCGTCTGCAT	Monomorphic
M10_SSR264	ATCGAAACAGCCAAAATCGT	TGAACCCTCCCTCACATCTC	Monomorphic
M10_SSR284	GCACCACTTTCACTGCAGAA	CTACCCCAGACCAAAAAGCA	Monomorphic
CN_SSR3	ACGAGCGATAGGGCTGAGTA	CGTCCTATTGTGTTGGCCTT	Monomorphic
CN_SSR5	CGAGTGCTTTAGCGTCACTG	GGAGCACATTCCTCATCGTT	Monomorphic
CN_SSR7	CATCGCAATGAGTGCAGAGT	GCCATTTGAATCCAAGGAAG	Monomorphic
CN_SSR9	TCCTTAGCATTCGAGGGATCT	ACGGCACTTATGGAGAGTGG	Monomorphic
CN_SSR11	TCACTTCGAGCTCATCAGGA	CACTTCCCTCTCAGGCAGAC	Monomorphic
CN_SSR12	TCTCGTGGCATCATTAGCAG	AGCAACATCAGCAGATGCAA	Monomorphic
CN_SSR49	AACTCAAATTGTTGCCCAGC	TGTTCGATTTTTCGTTTCCA	Monomorphic
CN_SSR52	TGCATTTTTGAGTGTGGATACAG	TGTCAGAGCAGCTCAAAAGC	Monomorphic
CN_SSR53	CCCAGTTGAGCTCATTGATGT	CGGTTATGGAGGATTTGGAA	Monomorphic
CN_SSR64	TTGAGCTTCCATTCCCTTTG	TGGTGATGGTGATGAGGATG	Monomorphic
CN_SSR69	CATTTCTTCATTCTTTCAAACACA	CAGGGAAAACAAGAGATGGC	Monomorphic
CN_SSR75	CAACCCAGCCTTACGTTTGT	TGGCAAGCAGAGAGGAAAGT	Monomorphic
CN_SSR81	GCCTTACCCACAAACCAAGA	AAAATCCAAGAAAAAGAAAAGTGC	Monomorphic
CN_SSR86	CTTTCGACTTGGGAGTGAGG	GAGAGAAGACCCACCACAGC	Monomorphic
CN_SSR90	ATAACCGGAATATGGGGCTC	GCTGTTGCTGCTGTGGATTA	Polymorphic

CN_SSR95	TAGAAACCAGTTGTGCCGCT	AGCTGCCTGATCACCTTCAT	Monomorphic
CN_SSR97	ATTCGCGGGTCTTTCTTTTC	GGACGGTGGACCCTACACTA	Monomorphic
CN_SSR103	CGATTCTTTTGACTACAAGCCC	TAACCCATTTTCGATGGCTC	Polymorphic
CN_SSR113	TCACATTGTCAACTGGAAACAA	GCTTACGTCATTCTGGAGGC	Polymorphic
CN_SSR118	CCATTCTCATTTCCTTTACCACA	TTGCCACGAGGTTCTTGAAT	Monomorphic
CN_SSR120	ATCTCGTTATTCTCGCCCCT	AAGGCATTACCAGTTGGTCG	Monomorphic
CN_SSR137	GCGACCTGAAAGCAACTCTC	GCCGTCTAGGGAATGTGTGT	Monomorphic
CN_SSR150	TGCAGTGAGAGATGGGATGA	CAAGTTTGCCTCCAAGCATT	Monomorphic
CN_SSR158	AGGCTCAAACGCTAAGCAAG	GGCTCGCAGAAGAAAGACAC	Monomorphic
CN_SSR170	CTCTGATCTGAGGACCGAGG	GCGGCACATTATCACTTCCT	Monomorphic
CN_SSR177	ACCTGCCCCGATGAAATACT	GGAGTCGAAGAACGTTGAGC	Polymorphic
CN_SSR185	TCTTGTGGCCATCAGAAGGT	TAAATGACGGGCTTTATGGC	Polymorphic
CN_SSR191	GAAAAACCCCCTAACTTGGC	GCTCAATCGTTTCAATCCGT	Monomorphic
CN_SSR199	GGCTACAGCCAATGCTCTCT	CAACAAAGGATGCTGATGGA	Polymorphic
CN_SSR203	TCCACCAAAAGCTTTGGAAC	ATTCTTCTGCTCGGCATCTG	Monomorphic
CN_SSR206	TGCATGCAAAACTGATGAAA	AAACGTTTCTGATGGCCTTG	Monomorphic
CN_SSR213	AATTTCAGCTTCAGCTTCGG	ACCAAAGGTAAACGTGGTCG	Monomorphic
CN_SSR219	GCATTGGGGTTGGCATAG	GGGTTGTTGGTGTGTTGATTC	Monomorphic
CN_SSR223	TTTCAAGAAAAAGAAAGGGTCTT	GGCCACTGAGCAGTATCCAT	Monomorphic
CN_SSR226	CGTGGTATTTCAGTGGGGAT	GCCTCCAACGAAAATCTCAA	Monomorphic
CN_SSR227	CCTCCATCAAGAACGACGTAA	AAAATGGCTGCTGCTCAGAT	Monomorphic
CN_SSR232	AGATTGCTCCAATTCCATCG	TGGCTTTTATCTGCTTTGGG	Monomorphic
CN_SSR242	GGCAAGTACAGGCCAGAGAG	CCTCTTCAGGGTTAATGGCA	Monomorphic

CN_SSR271	AGCTCAGGAGGCGTGTTG	AATTGCCTCCATGACGAGAG	Monomorphic
CN_SSR274	TCAAGTGAGACTGTAGTGGAGAAGA	CAAAAGCTGGCCTGAGAGAC	Monomorphic
CN_SSR276	TTGTGGTGGTTGTGGAATTG	GCAGTGGAAAAATGCCAAAG	Polymorphic
CN_SSR280	TTTACAGGTGGCGGGATAAG	ACGAGGGAAGAGGGAGGTAG	Monomorphic
CN_SSR282	GGATCTAGATGCTTCCCTGCT	AACTAAGCATTGGTCGTGGG	Polymorphic
CN_SSR285	CAACATGTTCATGTCGGCTC	TTGCATGTTACCAGGGATGA	Monomorphic
CN_SSR289	TTCAACAGCAGCAGCAACTT	GATGTTGCATTTGTGGAAGC	Monomorphic
CN_SSR292	GCCATTTGAATCCAAGGAGA	GGCCTAGAAGGCCATAGTCC	Monomorphic
CN_SSR300	GGACTGCCTTTTTAGCCACA	CCGAAACATTTAGCACCGTT	Monomorphic
CN_SSR307	CTCTTTGCCCTTTCACAAGC	GTGAAGCAATGGGTTGGATT	Monomorphic
CN_SSR317	GAGATCGGAAATGCCGAATA	GCCATAACTGAGGTTGCCAT	Monomorphic
CN_SSR321	TGATTGCCATGAAAATAGAATTTG	AGCAACACAAACTCCCAAGC	Monomorphic
CN_SSR323	TCCCCTGAAGAAACTGGAGA	TGAACAGAATCGACTCACCAA	Monomorphic
CN_SSR324	CTCGGTGAATGAAAGGAAGC	TGCAATTGTGCAAGTTGGAG	Polymorphic
CN_SSR325	AGTTTTGCTTCTCCGCTGTG	TTGGTTCATTTTTCACGTTTG	Polymorphic
CN_SSR326	GCACTTGAGAGACCCACCA	TGCACTTAACCAACCAACCA	Polymorphic
CN_SSR327	GCATTCTATAGTTCGGGCCTA	TTCATCCGCCATAAATTTCTTT	Polymorphic
CN_SSR328	CTGATGCGTCAAGCTTTTCA	CGTGTGATTAAGCCTACAGTGC	Monomorphic
CN_SSR329	GAACTAAATTCCTGCCCTTC	AAATAGTTTCATCCGCCA	Polymorphic
CN_SSR330	TTCATCGAGCTTGCTGTTAATC	TCCCTTCTCTAGGGAGGAAAA	Polymorphic
CN_SSR331	AAATTCCATGTGACTATGCTTTTTG	TTCATCAGGAATGGCTGACA	Polymorphic
CN_SSR332	AGGATTGAAATTGAAGTTATGG	TCCTTTAAGGGAATAAAATAGG	Polymorphic
CN_SSR333	GCAAACTTGATTGATGGTATT	TTGCTCTTATTACAAGTCTCCA	Polymorphic

CN_SSR334	CAAAGCTAACGTAAATGAAA	TCACCCTATTAGCTCCAAACT	Monomorphic
CN_SSR336	GTAACCCTAGCTAGCTCGAAA	TCCATCTTAATATTCTCCTTTCA	Polymorphic
JP_SSR4	ACCAGGATGCTGAGACTCGT	CTCCTGAAGGGGATAGGGAG	Monomorphic
JP_SSR10	GGAGAGCTCGACTCAGGAAC	TCTGCACTTTTTGGTTTGGA	Monomorphic
JP_SSR22	ACTACCATTTTCCTGTGCGA	ATGCATATAAGAACAAGAAACACTGA	Monomorphic
JP_SSR26	CTTCGAAGCCAATGGATGAG	CGTCAACTACAGGTCCTGCTT	Monomorphic
JP_SSR30	AGTGGTGGAGTTGCTAGCGT	AGAAGGGAGTGTCCAACCTG	Monomorphic
JP_SSR32	CCGATAATGAGGTCAAATTACAA	GTACTTGCGGTGGAAAATGG	Monomorphic
JP_SSR35	TCACGGTAAACGAACAAGCA	CCCCTTTCCACTTTCACAAA	Monomorphic
JP_SSR39	TGCTAGTGGTTTCTCTCTCCTTTT	TGGGCTTTCTTGGTTTGTTT	Monomorphic
JP_SSR40	CAAGAAAAGCACCCTTACCG	AAGCGTGGTCTACATACGCC	Monomorphic
JP_SSR41	GATTGGTGGAAGCTCGTGTT	CGCCCTTTCATCATCGTT	Monomorphic
JP_SSR46	TGCGCTATGAATGTAGCAGG	GAAAACTGGGCAAACCAGAG	Monomorphic
JP_SSR60	GAGTAGCCGATCCAGGTTCA	CTTGTTGTTCACTCGCGTTC	Monomorphic
JP_SSR63	CCCGTCCAATCCTCTCTTCT	TCCGATGACCAAGCTCCTAC	Monomorphic
JP_SSR79	GCTGTTGCTGCTGTGGATTA	CTCCTTTACAAGCTGGTGGC	Polymorphic
JP_SSR82	CCCGTCCAATCCTCTCTTCT	CCGATGACCAAGCTCCTACT	Monomorphic
JP_SSR84	CCGAAGTTGTCGGTTTCATT	AGGCAGGAACTGAACTGGAA	Monomorphic
JP_SSR107	GGCAGTGAAGCCTGTCTAGC	ACTGGAGCCAAGCTTGAGAA	Monomorphic
JP_SSR121	TTAACAGCAGGCAACAAACG	CGTTCTTGTGCCAATAGAGGA	Monomorphic
JP_SSR135	CATCAACATGGGATGGGACT	AGAGAACTGGCCATGCAAAG	Monomorphic
JP_SSR143	GCAGTTCTCGGCTTTTGTTC	CATCCCTCTGACTCGCTTTC	Monomorphic
JP_SSR184	AAGCACTGGAACTTGCAAAGA	TCTCAATGCCAAAACAAGCA	Monomorphic

JP_SSR204	GGACAAGCTCAATACCGGAG	GCAGGCATAAAGTTAGGGCA	Monomorphic
JP_SSR217	TGAAGAGGGGTTTGTTGGAG	TCCTTTTGCTCCTCAGCCTA	Monomorphic
JP_SSR247	CCATTTGATTTGATGGAGGG	CTCACAAGCCCAACTCAATG	Monomorphic
JP_SSR254	CGCGCGTATATAGCGAACTA	TACCCAGCTGTGGAGAAAGC	Monomorphic
JP_SSR273	TTCGACATGGTTGGTAGCAG	GGTTTTGGGAAAGGGAAAGA	Monomorphic
JP_SSR276	TCACTCTGTTTCCTACGGGG	AAGGCCAAGACCGATGTCTA	Monomorphic
JP_SSR306	CAATCAAAAGCACCAGCGTA	GCATGCAATTAGCCAACAGA	Monomorphic
JP_SSR317	GTGCCCATTTCACGGTAAAC	CCCCTTTCCACTTTCACAAA	Monomorphic
JP_SSR351	CCTCATCTGCCAATTAAAGC	GTTCAGAAAGCAGGCTCGTC	Monomorphic
JP_SSR364	GGGATGAGGCAGATGCTTAG	TCTTGTGCTTGCTGATCTGG	Monomorphic
JP_SSR393	ACAGTCATTGACAAGCGCAC	CACTTCAGCCGAAAAACACA	Monomorphic
JP_SSR406	TCAGGCTCTAAGCCACCATT	AAGCAAAGCTCTCAGCCATC	Monomorphic
JP_SSR417	TGGCAATAACGCATATCTAACG	AGCGATGATTGATGTGCAAG	Monomorphic
JP_SSR449	AAGCGTGGTCTACATACGCC	CAAGAAAAGCACCCTTACCG	Monomorphic
JP_SSR456	TCTCTATTCCACCAAACCGC	TCATGGATGCTTGTACCTGG	Monomorphic
JP_SSR466	CATCAGCCAGTCACATAGCAA	GGCTGCAACTTTGGAAGAAG	Monomorphic
JP_SSR507	GCTCTCTTAACTTGGCGCAG	CTATGAGCAAAAGGTTCGGC	Monomorphic
KL_SSR1	TACAGGGTCACAGCAGCAAG	AGTTTGTGGCTGAATCTGGG	Monomorphic
KL_SSR2	GTGCGTTGGATGGAAAGAAT	GCCCATACCCATAACTTGGA	Monomorphic
KL_SSR5	ACAACCAAATCCGACTGAGG	AACACCAGCAATTCCCACTC	Monomorphic
KL_SSR9	GCGGAAATGGGTATTTTCAA	GGCCTAGCATACATCATGGG	Monomorphic
KL_SSR10	ATGAGGCTCCGATGATGAAG	CCTCTGCTCCAAGTGCTACC	Monomorphic
KL_SSR11	TGAGGGATGCTATTGAAGGG	ACCATCACCATATCCACGGT	Polymorphic

KL_SSR12	GTGAGCACAGCAGCTCAGAC	GTCCTATACCAACGTTGCCG	Monomorphic
KL_SSR15	GGCGTGTGGAACTTTCTCAT	TAACTTGGACTCCTACGGCG	Monomorphic
KL_SSR16	AATCGTAACTGGAGATGGCG	GACACGAAGCCACCTCTCTT	Monomorphic
KL_SSR17	CGGGCACCTTCTAATGATGT	GTGCAGGTCCACCATAACCT	Monomorphic
KL_SSR18	CAATGAGCAGTTTGTAACCGAA	CTGGATCAAAGACCGCAGTT	Monomorphic
KL_SSR22	ATAGGGCACAAAGATGGACG	AGATTTCTCGACGGCTTCAA	Monomorphic
KL_SSR25	GGTTGGTGGTTCGAAAGAAG	GACGGCAGCTTCCTAGATGT	Monomorphic
KL_SSR26	GCTGCAGAGAGGGAGACAGA	GCCATTTCTTTCTTTTCTTTTATTTC	Monomorphic
KL_SSR29	AGGGACAGCTAGTTTGGGGT	CTACCCAGTCATGAAACCGA	Monomorphic
KL_SSR30	AATGCAGAAGAGACGCCATT	AGTGAGGATCATGGGCAGAG	Monomorphic
KL_SSR35	CGCGGTTAGTGTCAGTAGCA	ATGGAAGATGCGATAGCCAG	Polymorphic
KL_SSR36	GACGACCACCACGTAGCAC	TGGATCGCGACTCCTCTACT	Monomorphic
KL_SSR37	TGGTTGCAACATCTATCGCT	ATGACATCCGATAATCCCCA	Monomorphic
KL_SSR38	AGGCCATGATTTCATTCGAG	AGTAGAAGCCGCAGCAGAAG	Monomorphic
KL_SSR39	CCACCATCTCCTCCTTCTTCT	TGAAGATCCTACTGGCCTGG	Monomorphic
KL_SSR40	GCGCCACCTCCTCCTCTT	GAGTTGCAACGAAGCTGTGA	Monomorphic
KL_SSR44	GGGGGTTAAGTTTATTGCTTCTT	ACAAAGGCAGGCACTTATGC	Monomorphic
KL_SSR47	AACACTGCCACCGACAACTT	TGAAGATCCTACTGGCCTGG	Monomorphic
KL_SSR48	TGAGGGATGCTATTGAAGGG	ACCATCACCATATCCACGGT	Polymorphic
KL_SSR53	GTTCAGTGTCGACAGCATCG	ATCGACGTGGAGGACGAA	Monomorphic
KL_SSR72	AACTGTCCTGATTGCGGAAC	TTAACATAGCAGCAGTGGCG	Monomorphic
KL_SSR74	TTCCGTCACCTTGATCACAA	TCCTATTCCACCTTGCTGCT	Monomorphic
KL_SSR78	CACTACACCCTCTGGCTGAA	CCCAATCACAACCACATCAA	Monomorphic

KL_SSR80	GAAGACGAACTGGAGGTGGA	CGCTAGAGAGAGAAAAGCGG	Monomorphic
KL_SSR83	GATTAAGCCCTGGAACACCA	GTTACCCACCATACACGCCT	Monomorphic
KL_SSR88	GAAAAGTTTTTGGACAATGCTC	ACTCGCCACAAACCTGAATC	Monomorphic
KL_SSR91	ATTTGGTGATCGTGATGGGT	ACAAATCTTCCGCTTGCACT	Monomorphic
KL_SSR92	CCCAACTCTCTTCTCATGGC	CGGTGAAGCTACTAGGGACG	Monomorphic
KL_SSR93	GCTGTGACTTCAGGTAGGGC	TACGCCATCGTCACTTTGTC	Monomorphic
KL_SSR97	GCCACCTCTTCCTCCTCTTT	GAGTCCCAATCAACCTGGTG	Monomorphic
KL_SSR106	GCTAAGCTATTCATCAAATCAAATCTC	CATTTTGTGTGGTTTTGCCA	Monomorphic
KL_SSR110	ATCGCAACAGAGACGTCGAG	GCACTTTTCTTTATCTCCGCA	Monomorphic
KL_SSR115	CACCTACCATCCTTCCGAGA	TCCTATTCCACCTTGCTGCT	Monomorphic
KL_SSR118	TAGGGGCAGTATCTGGAAGC	ACAAACGAGGCTGAAGCTGT	Monomorphic
KL_SSR124	AGAAATCTCCATTCCGCACA	ATGCAAGATTGAGCAGCAAA	Monomorphic
KL_SSR131	TCTTGTTTTTGCTGTTGCCA	ACATCCGATAATCCCAGGTG	Monomorphic
KY_SSR3	AGAGACATGCTCAGCAGCAG	GACATCATTAGCAGCAAGCG	Monomorphic
KY_SSR12	GGAATCATACAGCCAGAGCAG	GAGTTTTTCTTCATCGGCCA	Monomorphic
KY_SSR16	CGCGGTTAGTGTCAGTAGCA	ATGGAAGATGCGATAGCCAG	Polymorphic
KY_SSR17	TTGACAAATGTGAAAGCCCA	GGTGGTGGTTTGGTTATGGA	Monomorphic
KY_SSR30	GAGGCCCTGGTAAAAACTCC	TCACATCACTAACCCAAGCCT	Monomorphic
KY_SSR36	TCGTGGAGAGTCTGTCTTGG	CTCAAAAGGCGGTTCTCTTG	Monomorphic
KY_SSR37	GTCTACAGTGTCCGGCGATT	CAACCGGTCGACTCCAGTAT	Monomorphic
KY_SSR51	ATGTCTGTTTGGGTTCAGCC	TCGATTTCCGGGTCTTAATG	Monomorphic
KY_SSR52	AATCCATTAATGCCCATCAC	TCTTCTCGGTCAAACCGAAC	Monomorphic
KY_SSR57	TTGCTATCATGCGCTGTCTC	AATCTCCGTCAACGTCCATC	Monomorphic

KY_SSR58	CAAATTGTCCATACACACCCA	ACTCCATCTTCTCCCAGGGT	Monomorphic
KY_SSR69	CTAGGAGATTCAGGTTGCCG	CCACATACCACAAAAACCCA	Polymorphic
KY_SSR82	CCAAAGGAGGATCATCCAAA	CAGGCAGCATTGGAGTAACA	Monomorphic
KY_SSR86	GCTTTATGGCAGAGACTGGC	CAGCAGCAAGTTTTCCAACA	Polymorphic
KY_SSR93	AGATCATGACCTCCACCAGC	AGCAACAACAGAGCTGCAAA	Monomorphic
KY_SSR96	CCACAAAAACCCACTCCTCA	TCAAATGGAAAATGGGGTTC	Polymorphic
KY_SSR108	GTAAGAAAAGCCAACCGCTG	TCACGGGTACACCTCCATTT	Monomorphic
KY_SSR112	GAGTTACAGGCCAAAGCCAG	TCTCGAACTCGCCATAGCTT	Monomorphic
KY_SSR127	ACATGACTTTCAAGCGAGCC	AAAGGAGGAGGCAATGGAAT	Monomorphic
KY_SSR128	TTTCCTCCATTGAGCACCTC	CCTCTCTGCTCTGTTGGTCA	Monomorphic
KY_SSR136	GACAGCACCTTCACGGTTTT	ACTCTTCACACCGTCTGGCT	Monomorphic
KY_SSR152	GCGTTCCAGTTGATGGTTTT	AACTCCACCATCCTATCACCA	Monomorphic
KY_SSR163	TCTTTGTCTGTCAAAAACCTCC	CAGCTGGGTCAAGAGACTCC	Monomorphic
KY_SSR165	ACAGAAGGGTCGGAAGGTTT	GCCTGCGCAAACATACATAA	Monomorphic
KY_SSR170	GAGAGAACGCGCAAGAGAAC	TACAGCGATTGGCTGAGATG	Monomorphic
KY_SSR171	CACCCTGGGTAAGGAGGAAT	GAAAGACAAGGGGAAAGAGTGT	Monomorphic
KY_SSR181	CCTCTCTCATTTCTCATTTTTGAA	GCAGCTTCATGACCAAGGAT	Monomorphic
KY_SSR182	CCCAGGAGCACCCAGTAATA	TGCTGTGGCACTAATTGAGG	Monomorphic
KY_SSR191	GGCGATTCTATGGAGGAGTC	GGAAATTTCTCACTCGTCGC	Monomorphic
KY_SSR196	CCAAGCTGCTTCCACACTCT	GTTCGACTTCGTGGCATAGC	Monomorphic
KY_SSR219	CATGAAGGTGCATCAGTTGG	TCTTGTCGTCACAGGAACCA	Monomorphic
KY_SSR221	GCCTGCGCAAACATACATAA	ACAGAAGGGTCGGAAGGTTT	Monomorphic
KY_SSR247	AGGTTACCCACCATACACGC	GATTCCCCTAACCCCAACAT	Monomorphic

JM_SSR8	ATTGGAGAAAGGCACATTCG	ATGCAACAGCAACATCAAGC	Monomorphic
JM_SSR25	AGCTTTCATCTTGGAAACGG	AAAAGCAGGGGTCTCCTCAT	Monomorphic
JM_SSR32	CAAGAATTTGATCAGGAAACGA	ATCAAGCACGTGCAACACTC	Monomorphic
JM_SSR69	GAACTGACGCCACTCCATCT	TATTCGGCTTTCATTGGGAC	Polymorphic
JM_SSR81	AAGCACATGGAAAAGCATCC	TCCTCAATTCCTTTTGTCCC	Polymorphic
JM_SSR83	GACCAAAGTTAAAAAGGAAAAACG	GACTCCTCATCCTGCTTTCG	Polymorphic
JM_SSR92	AGGAGGTGGAGGAAGAAGTGA	CGCCAGATAATTTTGGTGCT	Monomorphic
JM_SSR97	CCCAGCAACTGAAACATTGA	TGACAAGTGGACTTCGTTCG	Monomorphic
JM_SSR98	CGATCACTGGTGGGATTTCT	GCACAGCAATCACAGCTTCT	Monomorphic
JM_SSR99	TCCCTTTCTGATATCGACCC	CTCTCCAAAGGATGCTCCAG	Monomorphic
JM_SSR116	GCCAAGCTCAAAATCCTGAA	CAGAGAGATGTTGGCAATGG	Monomorphic
JM_SSR117	AACATCTCTGGCGACTGCTT	TGCAGCTCCATACCCTCTCT	Monomorphic
JM_SSR118	CCTCAACTGGCTTTGTCTCC	CAATAAGCCCCCTCTCAACA	Monomorphic
JM_SSR119	CATTACCCATTGCCCTTTGT	CGGTAATATTGGGGCAAATG	Monomorphic
JM_SSR149	TCTGCAGAGGCATCTGTGTC	CTGGCAAGGTCAGGGAATAA	Monomorphic
JM_SSR153	GCTCTGAAGACGAAACGGAG	CACACCCTATCTTCGCCATT	Monomorphic
JM_SSR157	TAAGGCTTGGGGAAAACACA	TCCAAATTCAAGGACTCCAGA	Monomorphic
JM_SSR158	GCATGCTGTCCACCTTACAG	TGCCACGTGAAAATATGGAG	Monomorphic
JM_SSR165	TCTCCACAACGGTCATGATT	GCAACTATTGTGGGCTTTCG	Monomorphic
JM_SSR169	TCTTGAGCAAGACGGCCTAC	CAGGAATCGGAAGCGATAAA	Monomorphic
JM_SSR194	AATGGAGGAGTTGGTATGCG	GCCAGAACACAAACACCAGA	Monomorphic
JM_SSR196	CCCAGGTAACAATTCCAGTTTC	TACATCATCAATGCGCCACT	Monomorphic
JM_SSR201	CGGCAGTATGAGCTGTCAGA	ATCCTGACTGGGAGCATTTG	Monomorphic

JM_SSR212	AGAGATGCTTGTGTGAGGGC	GTATGGTGTTTGGGCGAAAT	Monomorphic
JM_SSR216	GCCTGGAAGTGATTTATGGG	AATTCCATTCCAAGAATTGGC	Monomorphic
JM_SSR223	CCACCACCTTCCTCACATCT	GTGGGGCTGCAGAATATGAT	Monomorphic
JM_SSR228	CTGTCGCCACACCTCCTAAT	AACGGTGGCTTTTTATGGTG	Monomorphic
JM_SSR239	TTTGCACAGCTTGTTTTGCT	GAAGGAAGCCATGTGTGTTG	Monomorphic
JM_SSR248	GCAGAGGTTTTCCTGTCCAT	TGAAGAGCAATCAAGATGAGGA	Monomorphic
JM_SSR262	GCAACTTCAGATGCAACAGC	GCGCAAAAGAGGATCATTTC	Polymorphic
JM_SSR265	TCAACAGCACCAGTCAGCTC	TCGAGGAGGATTTAGTTGTGG	Monomorphic
JM_SSR269	GCCCTTCCAAGTACTGTTGC	CCACGAACTCCCCTGTACATA	Monomorphic
JM_SSR271	CCGACATGATAAGTTCTTGAGC	GATTGCGAGATAATTGCCCT	Monomorphic
JM_SSR280	GTTGCTGCTGCGACTCATA	TGGGGCACTCGAGTATAACC	Monomorphic
JM_SSR281	TGCAAGTTACCTCCATTTCATTT	CCTCCCTACAGCGAAGATCA	Monomorphic
JM_SSR287	TGTGAGGCTCAAACAACCG	TTCTTGGTTCTCGGATTTCG	Monomorphic
JM_SSR289	GCATGCCTTAACCTAACATCC	TCGAGTGTCAGCAGAGGAGA	Monomorphic
JM_SSR293	AGCAACGCATTCAATCTCTCT	TTCAAAACCTCACCCAAAGG	Monomorphic
JM_SSR299	CGTTGACCCCTTGTCAAAAA	TGCATTTCTGTTTCTCCTCG	Monomorphic
JM_SSR303	TCCTAATCTGTGCAGGAGAGC	TATGGAGACGTGTGGCTTTG	Monomorphic
Jcuint002	GATGTGGGATGCTGAGTGGTTTGA	ATTCTGAAGGCGGTGAGTGGTGAG	Polymorphic
Jcuint028	TTATCCGCCACAAACTTGATTATG	ACACCAAATTAGTCTTGCTTTCCTT	Polymorphic
Jcuint036	TTATTGGGTTGCGAGCCTCTTC	TTGCCTCCAAAGTCAAAAAGTCAC	Monomorphic
Jcuint038	TGCACGAACCGCAAGTCT	AAACTAATTCCCTGGGATTTTACA	Polymorphic
Jcuint039	GGGTCGGTATCAATTTATGACTA	TTACCTGGGCATTCAACAAT	Polymorphic
Jcuint044	AAAAATGGGAGAAATAAACGAAATA	CGTAGGAGCTGTTGAGCAGAGT	Polymorphic

Jcuint093	TTGACATAAAGGCTAAAGCATCTC	TTCCTGCCTAAAAATTCAATGATA	Monomorphic
Jcuint359	AGGTGAAAATAGTTGAGTGAGATTAG	AAATTACTGCCGCCTTCTTAT	Monomorphic
Jcuint047	TGGTCCTATGGCAGGCAAAATA	AATTGGCATGCTAGCCCATAGTC	Polymorphic
Jcuint090	GTTGATGGCATCCACCTGAAAAG	TTCGCCACTCTCATTTACACCATT	Polymorphic
Jcuint143	GAATGCCCAAGAATAACCCACTT	CCACTTGGCCAGAAGGTAAGG	Polymorphic
Jcuint301	GTTTGTATTTAAAGTCTCCTTTTCTG	AATGCCAAAACCAAATATTCAA	Polymorphic
Jcuint311	GAAAGGGTGTAAGCTTCCTCAAC	TCAACCTTTCCCAACCCATTA	Polymorphic
Jcuint312	CTGCCTGCTGCACGAAAAGAT	CCCGGGGGCTGAAGGAT	Polymorphic
Jcuint349	GGTGCGAATTTTACAACACG	TAAGCAACATTTTATTGAAGTAAGC	Polymorphic
Jatr229	AATGACGCTGAGACGGAAAAC	GAAAAGCTGCAAAACCCTATT	Monomorphic
Jatr333	TGCACCAAATTACTGTTATCGT	TGGCTGGTCGCATTATTAG	Polymorphic
Jatr362	ACCACCAGTTGCTCTTGATG	AACAGTCCTCCCCTATTATGC	Monomorphic
Jatr472	CCAACAACAGCATTTCCTTTTA	TTCCACTTGTTTTAGCTGTTCTTC	Monomorphic
Jatr681	CCTAAGCGCTTGCAGAGAACCA	CATCCATATTCGCGCTGTCGTT	Monomorphic
Jatr691	ATCATTTTTGCCTCTTCTTTTGTC	GCCCACATGTGACCTTCCTAT	Polymorphic
Jatr722	AAGATTAAATTGGTGGTTC	AAGCTCTTTGTTGTAGTAT	Polymorphic
Jatr795	GCTGCATGTCTGGCTAAT	TTGTCGAAAGGAACTATTGT	Polymorphic
Jatr800	TGGTGGGTAACAAGGCAGATA	CGCGCACATAAAACTAAAG	Monomorphic
Jatr826	TCCACGAACTAAGTAAAAGAAATC	CCACGCCATAGAGACTGC	Monomorphic
Jatr839	CCCTTGTATCTCTGCAGTCTTTTA	AAGGTGCGTTTCTGTCCA	Polymorphic
Jatr859	TGGCCCGTCGAAATGAAG	TGATGGAGCAACTTAGCAGAG	Monomorphic
Jatr866	TGCCGACCGGAACAACAAAA	ATTAAAGCAGCTCCATCCCAGTCC	Monomorphic
Jatr883	CTCCGCTATCTCTGTCTCCA	TGACCTTGAGTAGCCATTGAT	Monomorphic
Jatr901	GAGCCTGCCAGAAATAAAATAACA	GCACGAACCGCAAGTCTCC	Polymorphic

Jatr971	CTGACTCGCGTGGCTACTG	CACTATTATCAAAAGCGAGAAAAC	Monomorphic
Jatr979	CTCTCATTGTTGCGCTGTCATCA	TACGGGGAGTCCACCATAAAACCT	Polymorphic
Jatr1010	TAGGCAACAAGGAAAAATAACG	TTGAATCAGGGGGAAAGTGT	Monomorphic
Jatr1035	CATTCCCTGTGCGCTACTCTC	TCATTGTATTGCCCTTGTTTATCA	Polymorphic
Jatr1048	TCTCTTTCACTGTGCCTCTTTT	GTATTCTTAGCCATTGCCTTTTT	Polymorphic
G25	CGGTGCACATGATGCCTAACC	CGCCATCTCCGCTTCATTGC	Monomorphic
G44	TGCTTAGGTGGAGGTTATGTGGCT	AGGGTTTGTTCATCGGTAAGGGGT	Monomorphic
G58	ACCTTTTGAGTTCTGTTGTTCGCA	CCATTGTTGGTGGAGGCGGTGA	Monomorphic
G80	AAAGTAGGGCTGCAGAATCTTTAAATAGCA	CCATGCCACAAAGAGACTTGAACTGA	Monomorphic
G141C	CGTGGCGATGTCCCGATGGT	TGCATTCTGCTCCGCTGCAA	Monomorphic
G144	AGTGTTGGGTCCAGGTGGTT	CTTCTGCCCAGGGCCCAAAT	Monomorphic
G262	GGTTGCAGCAATTCTTCAGCTCCCA	GCAGGAGGTGCTCCCCTCAA	Polymorphic
G268	TCTGACAGGTCACCAGAAAGGCT	CGGCGATCCAGATGCTGCTGA	Monomorphic
G269	ACCAGATTTTTCCCACCATGCATACCA	GCCAGGCGAGAGGTTGGAGG	Monomorphic
G270B	GGATACACCCCTTCTGTTGGCA	CACAGCACCAGACACTAGCCA	Polymorphic
G270D	AGACGTCAGAGAGATCAGGCACA	CTGATGCGCTCAAAGCCTGC	Polymorphic
G273A	TGCAAGACTGATACAATAACGGACG	GCAGAGCTATTACTCGTCTTTGGC	Polymorphic
G282B	TGATCGGAATTACCAATGTGTTAACGG	TCAGCTTTGATTGGATAAGCCATGTG	Polymorphic
G284	TCGGGTGGTGGTGATATGCAGT	TCTGGTGATGTTGTTTCGCGT	Monomorphic
NG285A	AGCTAAACCAGACAGACCCCCTGT	TGGCTGGATGTGGCTTGTCCT	Monomorphic
NG286A	CCCCCAACCCATGCGTGCTT	GCACCCACACGGCCACAGAA	Polymorphic
NG286D	TGGGATGACAGACCCCACCACC	TGGCCAGGCCTTCTGGAGAGT	Monomorphic
NG287B	GCACTATGTGCAGTGATGGGGCA	TTGCCTGCTTTGGCCCCCTG	Monomorphic
NG288C	TCCCATACGCTTTTGAGTACCATGT	AGTTGGTAATGGCATCACATTTGGA	Polymorphic

ND289B	CGGAAATGGTGACTCTGCACCG	ACCGCTGCCTCCAGTGACCT	Monomorphic
NG291	CCCATTCCTACACAATATACAAGCCAA	TCCCTTTTGACCAATGTCACTATTAGC	Polymorphic
JCT3	GCCATCAACCGTAGTCGCAAACG	CCAGCAAGGGGCTTGGCGAT	Monomorphic
JCT23	ACAGGGACCAGACCCAGGAAAAGG	AGGTCAGCAGAGGCGACGGT	Monomorphic
SSR-EST15	ACAACACCGTACGCGTCGCC	GCATCGAATCCTCCGTGACTATCACCA	Monomorphic
AG343	TGGTCTTATTTGTTGTGAAGGC	CATCAAGAGATTGGGAGACACA	Monomorphic
0_38	TCCACCAAAACTGAGACGAA	GGGCAACATCCTAAACCTGA	Monomorphic
0_1890	ACGGGAATGAATAGCACTGG	CCCACCGCATAAAAGAAAAA	Monomorphic
1_1967	CCAGCATTTGTTGTTATTACACTG	CACGTGCGAGGTAAATGAGA	Monomorphic
1_20	TTGTCAGGCAACTGAACACC	CCATCTCCTTGACGGTAGGA	Monomorphic
2_9	ACTGCTCTGCTGTGCTGTGT	TGTTTGGGAGCTAAAGGGAA	Monomorphic
2_1337	TTCATGAATGGCTGCTCTTG	CAAGTCGTGCATTTGCTGTT	Monomorphic
3_1294	CCAGGGAAGTGAACCAAAAA	CAACGTGTGGTTTGACGGTA	Monomorphic
3_6	AGAACGCATGAGTGCATTATT	GGGTAAATATGTGTTTAGCGGC	Monomorphic
4_1157	CACCAAGTATGAGGAGGAGG	AAATGTATCTCCCATTAGATTTGAA	Monomorphic
4_19	CGAGCAAAACTGAAAAAGGAA	TTCCCAATATTGCTGCTTTTG	Monomorphic
5_3	TGGTGCTTGTAAATTGAGTGC	AGGCGCAGTAAGCTGTTGTT	Monomorphic
5_1162	CCTTTTGAATACCATTCACCAAA	AGGTGTGACTTTGCCAATGA	Monomorphic
6_8	ATACCCCATTCCTCTGGTCC	AATACGGGTCCAGGCCTATC	Monomorphic
6_977	TTAGCCCCAGTCAAAGGATTT	TTGGTTGGTTGGTTCTCAAA	Monomorphic
7_15	TGCTGTTCATCCTTCAATGG	TCGAAGAGATGCACCAGATG	Monomorphic
7_937	CCTGCATGTGGCAAAGTTTA	GGTTCCTCCACAGCATTGTT	Monomorphic
8_1117	TCTTATGCGCATCGACTGAC	TTAACTCCACCTGGCTTTGG	Monomorphic
8_47	AAAAGCGATTGCATTTGTCA	CTCTCCATCTCCCCCTCTTC	Monomorphic

9_18	CCCAAGGAAAACGAAAATGA	GTTTTCTGCGCTTCTTGACC	Monomorphic
9_935	GGGTTTTGGAAAAACGTTGA	GTATGTCTGGCAGCGTTCCT	Monomorphic
10_909	AGTGGAGGGATTGATGTGGA	CACCCCATCAAGACCATTTC	Monomorphic
10_5	TTCCTAGATTTCAGCCCCAA	AGGACATCCCACTAATCCCC	Monomorphic
11_775	CCATTGGCAAACTCCTCAAT	TGCAGCGGAAATTAAGCTCT	Monomorphic
11_5	TGAAGAAAGGGCAAAAAGGA	TGCACTGCTGCCAAATTAAG	Monomorphic
12_6	GGTATCATTTCCCAAGTGGC	TTTGCATGGGATCTGCATTA	Monomorphic
12_861	CCACCAAATTCTTGTCAAAGAC	CGGTGGATTTAGCTAGGAAGC	Monomorphic
13_975	AGGCCCATAACGAACAACAC	GCCGAGTCAGAGGATTTGAG	Monomorphic
13_3	TCCAGATTCCTAGCTGATTATTGA	AATCATAATGCCCGACCTTG	Monomorphic
14_74	CCGCAGATACAAGCAAGGTT	CCCACCAATGTCGCTAAAAT	Monomorphic
14_857	CCTTGGTTGGGACTTGTTGT	GAGCCAAACACTTCTGGAGG	Monomorphic
15_84	ATCCACAATTGTCTCTGCCC	GGGCAAATTTAATGGTTAGGG	Monomorphic
15_708	GACGCAATCTGTCCAAGGAT	CTTCAACGCTCATCTCCCTC	Monomorphic
16_687	GGCTCCTCTCTCTGTTTGGG	AGCAAACAGATTCCGATTGAG	Monomorphic
16_10	CAGTTTCAATGGCTGCAAGA	GAAGGTGCGAAGGCTTATTG	Polymorphic
17_824	TTATGGAAATATGCTTGTTGGTT	TCATTACGTAGTATTTTAGCACGAGG	Monomorphic
17_84	GTGAGGTTAGAGCTCGACGG	CCCAATTTGAAACGCAAAAT	Monomorphic
18_22	AAGCGAACTTCACAATTCCA	GATTTCGCTAGCACCAGGTC	Monomorphic
18_830	TTCTCAAAAGGCAAAATGGC	TGTCTGCATTAAGTAATTTCTGAGC	Monomorphic
19_5	GGGTTTGAATTTGATGCAGG	CAGGTGCACTAACAAAACCG	Monomorphic
19_849	GTAGGGTTGCCTTCAAACCA	TGATTCCGACCCTCTCATTT	Monomorphic
20_8	GGAAGGATCGTTCTTCCAAA	CATGGAATCACACAAGGAATCT	Monomorphic
20_736	TCGGTGAAGAATGTCTGCTG	CACCAACCGCTGAAGTACAA	Monomorphic

21_790	TTCCTTCAGCCCAAGAAAAA	TGAGTTGGAATGGTGTGGAA	Monomorphic
21_71	AAAAAGGAATAATGGCTTGATTT	TGGGAGGACATACTTCGATCA	Monomorphic
22_10	GCGGTTGCTTGAAGACTCTC	AGGAAATCCCTAAGGGCTGA	Monomorphic
22_666	ATATCGGCATTTTTGTGGCT	AGAGCCAGGTAGTCAGTGGC	Monomorphic
23_728	CAAAACTGCGACGTTTCGAT	TTGGATCTTAGTTAAATGCCTTTTT	Monomorphic
23_8	AGGAATCTTGGGTGATGTGG	TTTGGTGGGTTTGAAAAAGG	Monomorphic
24_55	AACGGATTTGGTGATTGCAT	TTTCAGCTGCAAGAACAATGA	Monomorphic
24_513	TTCCTGAAAAAGGCAATGCT	ACCCTCTTGGGCTTTTGTCT	Monomorphic
25_17	TACAGCTCAACCCTCACACG	GACGACTCCGTTGATTCCAT	Monomorphic
25_756	TGTGGTGGGGTTGGTATTCT	CCATTTCCAGAAAAATTTCGAT	Monomorphic
26_577	TATCGGGGTCCCATTGATAA	GTCCCACGTGCATAGATCCT	Monomorphic
26_21	TGCATTTTCATCACTTTGCC	TGTCGTTTGTCGTTTGCTTC	Monomorphic
27_744	TGCACAATTGGACATTCATATTT	TAGAGGAGGCTCCGTCAAAA	Monomorphic
27_10	CCACTCAGCTCCAGCTTTTC	TGAGATGCATGCAAACAACTAT	Monomorphic
28_9	TTCCACTATTGCAGCTTTGC	TGACAACAAATAGCCACCGA	Monomorphic
28_666	TTGAAGCTCTCCAGCAATCA	CAATCTGAGCGTAAAAATACGTG	Monomorphic
29_34	GCGGTTCCTAAGATGGTTTT	AAAATCAGTTCCGGTTTGAA	Monomorphic
29_617	GCATCAAAATCAATCTCCCC	GGTCGAAGCATAGGAGAGGA	Monomorphic
30_4	AGCGTTACGAACGGAGGAG	TTTGAGTCGCAAATCAGTCG	Monomorphic
30_511	AAGAAACCAGCATGACAGCC	CAAGTGTTCAAAATGACCCAAA	Monomorphic
31_705	TCAGCCTCTTTCGAGTCCAT	TCCGAAGCTGTGGAAACTCT	Monomorphic
31_71	TCACGAGGCAAACACTGAAG	ATCAAAGCTGGTCTCCCTCC	Monomorphic
32_734	GGAATCCAAATGTGATGCCA	ATGCTTTGGCAATCCTTGTC	Monomorphic
32_8	TTGGTCTGCGTGGTTGTTTA	AGTGCATGCAGCACCTAAGA	Monomorphic

33_540	GCAATGTGGATGGTTCCTCT	TTCATCTCCACCATCAACCA	Monomorphic
33_2	TGGCTGGGTCTTAAGATTTCAT	TCGTGTGCTAAAAAGAAAGCAA	Monomorphic
34_544	CGTGGTAAAAGGAAGGTGGA	CATGGCCCCACTCTCTTAAA	Monomorphic
34_42	AACAGGGCATTTTGACTTGG	CATTGCCAATCCTGAATCCT	Monomorphic
35_4	ATTGGAGGAGGCCAGAAAAT	ACTAAATGATCCTTGCGCCC	Monomorphic
35_597	TCACAGTCTTCTGGTGGCAA	CAGTTCCCTTCCATTTCCAA	Monomorphic
36_545	ATGTACCCTTCCCCCTTCCT	TGGAAATTCTTGACTTGGTCAC	Monomorphic
36_9	CCCCTTCAAAATAGACGCAA	TGGATCGGCCACTTATTTTC	Monomorphic
37_7	TGAGCCCAAATTGTGACTTG	TAATTGCGGAACTCTGCTCC	Monomorphic
37_624	TGTCCCATGGACGTACCTAA	TTCAAAGTGTTCCTTTGATTTTT	Monomorphic
38_18	GATGGGTTGGTAGGCTGAAA	ATAGGTGATGGTGGAGGTGC	Monomorphic
38_368	CTAGGCTCCATTTGTTTCCG	CACTCACCATTCACCACCAG	Monomorphic
39_588	GGGGCAGTATGAAAATCCAA	GTAATCCGGGTTGACTGCAT	Monomorphic
39_10	TTGATTGCTGGCTTTGTGTT	AAACTCGTCGTGAGCCATTT	Monomorphic
40_632	GAGTTGATGGGGTTTTCAGG	GCAAGCGTGAGCATTAACAA	Monomorphic
40_18	GGGTAGAGAAACGCATGGAA	TTTTAATCAAGGAACCCGCA	Monomorphic
41_579	TGGATGCAACGTTACCATGT	TTGTTATTGCAAGTTGGCGA	Monomorphic
41_47	ACCAAACAACATCCACCCAT	TGAAGATGCAGTTGACTCCTG	Monomorphic
42_458	ATCGGTCAACCAAATTGGAA	TTTTCAGGTGAAGCTTTGAGAA	Polymorphic
42_14	ACCCTCATCACTTTTCCACG	TCAATCTCAACAAAATGGCAA	Monomorphic
43_458	TCTGCTACTCTGTCTCGCCA	CTGGAACCCTCTTCTTTCCC	Monomorphic
43_23	TCGATTTGAAACCGTGAAAA	AGACGGTTTTGAAATTGCTGA	Monomorphic
44_355	TGGTATCAGAGCAAGCATCG	GAACTCGAGCCCAAGAATGA	Monomorphic
44_31	CGTTGGACCTCCCATGTATC	AAGCAAAATTTTATGATTAGAATAAGG	Monomorphic

45_594	TCTTTCGGTGTGTGTCGTTT	CAATGGCTGTCGTTCTGAAA	Monomorphic
45_55	AAAGAGGACCACCAGCAGTG	GGTTGGCTTTCAGAGTGCAT	Monomorphic
46_55	GGTGACATGGTTGTGAATCG	CAGATGATTGATTCGGTTTGA	Monomorphic
46_591	ATAGGAAATCGAAAACCGCC	TGATTCTGTGGTGATCCGAA	Monomorphic
47_536	AAAAACCATGAAATGGCGAC	TTGCAAAAGTTCAAGTGGGA	Monomorphic
47_14	GGAAAGCGGAACAAAATGAG	GGACCTTCAACACCATCCAC	Monomorphic
48_581	TGTGTCATAGTTTTATCCTAGGTTTTC	TGAAACTTCATTAACGCTTCAAA	Monomorphic
48_23	AACGGTAAAGGTCGTGGTTG	CAAACCAAAGGAGAACACGTC	Monomorphic
49_7	TTCCAAAACTCGTTTCACCC	AAGGCATTACCAGTTGGTCG	Monomorphic
49_533	GGTGGTGTGTGGCTTATGTG	AAAAATGGTGCACTACCAAAAA	Monomorphic
50_96	GCCTGGTCACTGAGATCCAT	TTGCGCTGTTCGATCACTAC	Monomorphic
50_467	TTTGCATTTTCTTGAGATGGG	ACTGTTTGCATCTCCAAGGC	Monomorphic
51_34	GCCCATGAAGTTACCACCTG	TGGACATGAAGATGAAGGCA	Monomorphic
51_585	GCTCGACACCATTTTTCGAT	TGGCCTAGAAACCTCTGGAA	Monomorphic
52_555	GGAAATTCCTTTTGCCCACT	AAAAAGAAGGAGCCAGGAGG	Polymorphic
52_11	TTTGCAATTAGGGACCGAAA	GCGTTTAGAGCACGACCAAT	Monomorphic
53_542	CCACTCTCCAATGCCTCACT	CAGCTTCCAAACTAATGGGA	Monomorphic
53_10	ATCCCCTGCCAAACACATAG	GCCCCAAAATTCTTGCTGTA	Monomorphic
54_61	ATCAATGAAATGGCTTTCGC	CTCAGACGCCACAAGATCAA	Monomorphic
54_527	CCAACTCCCAATCACCAACT	GGACTCGGGCTTATGACAAA	Monomorphic
55_619	AATTGGAAGCCATGCAAGAG	TTTTGGGGATTTCATCAAGC	Monomorphic
55_75	TGGATGAAACCTGTGATGATG	TGGTTGAAGTCATCCCAAGA	Monomorphic
56_574	TTCAAACGATTTCGATTTATGA	TGAGGGATGCATGCAGAATA	Monomorphic
56_10	GGAAGCCAAATACATGGCAA	TCTCCAGTCCAAATGTGAAAA	Monomorphic

57_39	CGGCGCATTCTTCTCATATT	GAAATCATGGGGCCAGTCTA	Monomorphic
57_397	TGCATCCACAATTTCTTCACA	TGACAATGTGAATGCCTCAAA	Monomorphic
58_570	TTGTCAACTTTTTATTCCAATTTTT	TGGAACGAAGTTTCAACCTAAA	Monomorphic
58_29	GGTACGCAGGAACGAAAGAG	GAAAACCAAGCAAATCGAGC	Monomorphic
59_2	GGAATGAGCCATTGCAAAAT	TTTCGGCTTCTCTTTCTCCA	Monomorphic
59_648	GCCGTTAGGTGTTCTAATAGCTG	TCACAAGTGCTTCCACACAA	Monomorphic
60_553	CTTTTCCAAATTCAGGTGTTCA	ACATCACATCGCCTGCACTA	Monomorphic
60_3	CCACAGTTGGAAGAAGACCC	CCCACCTACCCTCTTCCTTC	Monomorphic
61_7	GCAAATTCTCCCATGATTGC	CATGTGCGAAATCTGTTTAGG	Monomorphic
61_586	TCGGATTGGAGGCTAACAAC	ATCCAGGCAACAAAAACAGG	Monomorphic
62_614	ATTCCGGTATGAACACTGCC	AAACCCCCGTGAAGAAAATC	Monomorphic
62_17	TTCAAAATTTACATTAAAAGCCAAA	TCATGACTCTCGGCTTGGAT	Monomorphic
63_474	GCACCAAACTAAAACGCCTC	CCGGAAAACAGGGACAGTAA	Monomorphic
63_8	AATAGAGGCAAAAGCGCAGA	AATCTCCTATCCTAGCAAACAAAA	Polymorphic
64_534	CATCACTGTCGTCATCGCTT	TTGAAAAGGGCAACTTAACAAA	Monomorphic
64_17	TATCTGTCGTTGGTGGCAAA	AGGGGTTCTGCATCATCATC	Monomorphic
65_443	TCCTTTCAACACTTGATAATTGG	ATGAGAGGCCCAATAAACCC	Monomorphic
65_85	TGAATTGATGCAATTTGATTGTT	TTCCATATATCATTAGAAACTCGAAA	Monomorphic
66_29	GGAATTTTTATGCAAAACGCA	CGTGCTTATCACTAACGGCA	Monomorphic
66_466	TCTCCTTCTTGCTTGGCATT	GGCTTTAACAGCAAATGGGA	Monomorphic
67_8	TTTGGGAGTACCTAAGGTTCCA	CATCACTGATCACCGACGAC	Monomorphic
67_325	GGATGAAGGTGAAATGGTGG	TGAGTCAGGACACATGCACC	Monomorphic
68_25	GAATTTCCTAAGGCCCCAAG	CTATCCACACACCAAAGGGG	Monomorphic
68_553	TCAACGCATAGAGTTGCCAG	GCCACTTGGTTGATTGAGGT	Monomorphic

69_474	ATTTTAACCCATTGGCCTCC	GGCTGGTTGCTGTATGTCAA	Monomorphic
69_32	GTGGATGTCTGAGGCATGTG	CCTTTCACTCGAGGCTTGAC	Monomorphic
